# Contribution of mammary epithelial cells to the immune response during early stages of a bacterial infection to *Staphylococcus aureus*

**DOI:** 10.1186/1297-9716-45-16

**Published:** 2014-02-12

**Authors:** Pauline Brenaut, Lucas Lefèvre, Andrea Rau, Denis Laloë, Giuliano Pisoni, Paolo Moroni, Claudia Bevilacqua, Patrice Martin

**Affiliations:** 1 INRA, UMR1313 Unité Génétique Animale et Biologie Intégrative, équipe «Lait, Génome & Santé», F-78350 Jouy-en-Josas, France; 2 INRA-Plateforme de Microgénomique ICE (Iso Cell Express), F-78350 Jouy-en-Josas, France; 3 Department of Health, Università degli Studi di Milano, Animal Science and Food Safety, 20133 Milan, Italy; 4 Quality Milk Production Services, Cornell University, 240 Farrier Road, Ithaca, NY 14853, USA

## Abstract

To differentiate between the contribution of mammary epithelial cells (MEC) and infiltrating immune cells to gene expression profiles of mammary tissue during early stage mastitis, we investigated in goats the in vivo transcriptional response of MEC to an experimental intra mammary infection (IMI) with *Staphylococcus aureus*, using a non-invasive RNA sampling method from milk fat globules (MFG). Microarrays were used to record gene expression patterns during the first 24 hours post-infection (hpi). This approach was combined with laser capture microdissection of MEC from frozen slides of mammary tissue to analyze some relevant genes at 30 hpi. During the early stages post-inoculation, MEC play an important role in the recruitment and activation of inflammatory cells through the IL-8 signalling pathway and initiate a sharp induction of innate immune genes predominantly associated with the pro-inflammatory response. At 30 hpi, MEC express genes encoding different acute phase proteins, including SAA3, SERPINA1 and PTX3 and factors, such as S100A12, that contribute directly to fighting the infection. No significant change in the expression of genes encoding caseins was observed until 24 hpi, thus validating our experimental model to study early stages of infection before the occurrence of tissue damage, since the milk synthesis function is still operative. This is to our knowledge the first report showing in vivo, in goats, how MEC orchestrate the innate immune response to an IMI challenge with *S. aureus*. Moreover, the non-invasive sampling method of mammary representative RNA from MFG provides a valuable tool to easily follow the dynamics of gene expression in MEC to search for sensitive biomarkers in milk for early detection of mastitis and therefore, to successfully improve the treatment and thus animal welfare.

## Introduction

Mastitis is an inflammation of the mammary gland (MG) commonly caused by bacterial infection. Despite extensive management practices, it continues to be an economically important disease of dairy ruminants worldwide, due to reduced milk yield, milk discarded after treatment and cost of veterinary services [1]. A new challenge today is to reduce the use of antibiotics and treatments by increasing the natural ability of animals to resist infection. This strategy is critically dependent on a better understanding of the host immune response at the early stages of infection. Indeed, the establishment, persistence, and gravity of infection are clearly dependent on the rapidity and effectiveness of the host response against the invading pathogen. Furthermore, clearance of bacterial pathogens from the gland is often governed by responses that occur within immediate hours after initial infection [2].

At the early stages of infection, the predominant defence strategy that is rapidly induced is the innate immune response. This response is ubiquitous, short acting and targets a range of different microorganisms [3-5]. Cells involved in this innate response are mainly represented by infiltrating neutrophils and macrophages, which are the first cells to be recruited at the site of inflammation [6]. They secrete cytokines, chemokines and use additional cellular defence strategies to kill invading bacteria [7,8]. However, little is known about the factors involved in this rapid recruitment at the site of infection. Hence, studies have increasingly focused on the mammary epithelial cell (MEC) since it is the first cell to be confronted with the pathogen once it has entered the mammary gland. There are more and more lines of evidence indicating a prominent role for MEC in the initiation of the innate immune response that triggers subsequent neutrophil infiltration into the mammary tissue, the activation of these innate immune cells, and an increase in milk somatic cell count [9].

However the MEC role has only recently been recognized essentially using in vitro approaches which allow examining the MEC specific contribution to the immune capacity of the udder without the immune cell expression. The effect of different pathogens including *Escherichia coli, S. aureus*[10], *Streptococcus uberis*[11], and bacterial cell wall components such as lipotechoic acid (LTA) or lipopolysaccharide (LPS) [12] were tested on different mammary cell lines in either primary isolates of bovine MEC (pbMEC) or MAC-T cells, an SV40 immortalized bovine MEC line [12,13]. These studies indicate that MEC respond robustly and rapidly to challenges with low levels of bacteria or bacterial cell component and show that MEC is able to generate a variety of inflammatory mediators such as cytokines, chemokines and host defence peptides (β-defensins). However, these reports underlined some limits associated with in vitro experiments and show differences between mammary cell cultures (pbMEC and MAC-T) [12]. Furthermore, cells are studied out of their physiological context and consequently, they do not properly reflect changes in gene expression induced by mastitis in the udder. Thus, a disproportionate increase in chemokines such as CCL5 has been shown between pbMEC and the whole mammary gland [14]. In addition, there is only one study profiling the extents of global changes in the transcriptome of pbMEC during the early stages of infections with *E. coli* and *S. aureus*, reflecting in part the kinetics aspects of immune response regulation [15]. Consequently, the role of MEC in the initiation of the innate immune response, in their physiological context and in the very early stages of infection, remains poorly defined.

Recently, we have reported [16] that substantial quantities of high-quality RNA, coming from MEC, can be isolated from milk fat globules (MFG). This non invasive method allows easy and repetitive sampling without damaging mammary tissue, providing a significant improvement and a valuable tool to assess gene expression of the mammary secretory epithelium during the course of an infection. Furthermore, with the availability of microarray technology, which makes it possible to examine complex interactions between host and bacterial pathogens [17], it has become feasible to determine the expression of multiple genes simultaneously. We successfully applied this approach to follow the response of MEC early in the infection process of the goat mammary gland.

We report here that MEC rapidly react (a few hours after the experimental infection) by secreting the chemotactic factor interleukin-8 (IL-8), which is known to be one of the major mediators of the inflammatory response involved in the recruitment and activation of immune cells, including neutrophils. Furthermore, we provide strong evidence that MEC specifically expressed several acute phase proteins (APP) such as serum amyloid A3 (SAA3), pentraxin 3 (PTX3) and alpha-1 antiproteinase (SERPINA1). This work also indicates that the production of different APP by MEC, easily detectable from milk in the early stages of IMI, could provide sensitive biomarkers for early detection of mastitis and therefore, to successfully improve its treatment and thus animal welfare.

## Materials and methods

### Animals

Five healthy Alpine goats, at the early peak of lactation (30-40 days of milking), at first parity and without intra mammary infection (IMI) were selected and housed at Centro Zootecnico Didattico Sperimentale of Facoltà of MedicinaVeterinaria (Università degli studi di Milano). Goats were monitored for IMI throughout the lactation period with 3 weekly half udder milk sample analysis before challenge. Bacteriological analyses were based on procedures previously described [18]. Briefly, ten microliters of each milk sample was spread on blood agar plates (5% defibrinated sheep blood). The plates were incubated aerobically at 37 °C and examined after 24 h and 48 h. The colonies were provisionally identified based on Gram stain, morphology, and hemolysis pattern, and the numbers of each colony type were recorded. The representative colonies were then subcultured on blood agar plates and incubated aerobically at 37 °C for 24 h to obtain pure cultures. Catalase and coagulase production was tested for gram-positive cocci. Specific identification of staphylococci was made using commercial micro-methods (API Staph; BioMerieux, Italy). The infection status of milk samples was defined according to the procedures recommended by the National Mastitis Council (NMC, 1987) and IMI was diagnosed when ≥ 500 cfu/mL and 1 to 3 colony types were isolated. Milk samples from which many colony types or < 500 cfu/mL of any microorganism were isolated were regarded as contaminated or uninfected, respectively. At the moment of challenge, no infection in the udders was observed, as was confirmed by the absence of mastitis pathogens in foremilk samples tested for three consecutive days just before the experimental challenge.

### Experimentally induced mastitis and milk samples

**Figure 1:**
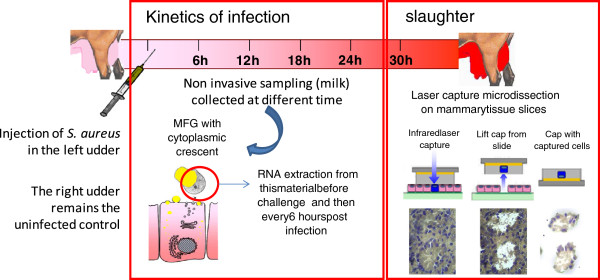
**Experimental infection workflow.** The left udder was challenged by *S. aureus* whereas the right udder remained uninfected as the control. Then every six hours, milk was sampled and centrifuged to extract RNA from Milk Fat Globule (MFG). At 30 hpi, goats (*n* = 5) were slaughtered and mammary tissue samples were taken for MEC capture experiments using LCM (total RNA extraction from micro-dissected MEC) and total RNA extraction from deep alveolar mammary parenchyma (referred to as MG). Finally, these different sources of RNA were analyzed using microarrays (only MFG) and qPCR.

Prior to intra mammary challenge, both half-udders were milked by hand and the teat ends were carefully disinfected with chlorhexidine. The left half-udder of each goat was infused with 1 mL (10^3^ CFU/mL) inoculum of *Staphylococcus aureus* strain DV137 which was originally isolated from a chronic case of caprine mastitis. The right half-udder, used as an uninfected control, was infused with 1 mL of sterile pyrogen-free PBS (Figure 1).

First, milk samples from uninfected right (Rx, T0) and left half-udder (Lx, T0) were collected before IMI with *S. aureus*. Three hours later, intra mammary challenge with inoculum of *S. aureus* was carried out. Subsequent milk sample collections were carried out at 6, 12, 18, 24 and 30 h after challenge from both right (uninfected) and left (infected) half-udder, taking care to avoid ribonuclease (RNase) contamination: cleaning the udder and teats with a clean linen impregnated with an antiseptic solution followed by a spray of RNAse Zap, drying with a paper towel (Kimwipes) and performing a manual milking with disposable nitrile gloves. For each animal and time point, 150 mL of milk were collected from each half-udder into sterile, RNase free tubes (3 × 50 mL Falcon tubes). Samples were immediately kept on ice prior to MFG collection.

The clinical scoring system to classify mastitis symptoms cases was as described for dairy cows [19]. In addition to abnormal milk, this system is based on measurement of rectal temperature, hydration status and clinical attitude. Severity of clinical signs was scored as mild, moderate or severe. A mild score was assigned when the milk was grossly abnormal and no other local or systemic signs of inflammatory disease were seen; a moderate score was assigned when the milk was grossly abnormal and there was firmness or swelling of the affected mammary gland, but none or only one of the systemic signs of inflammatory disease are seen. A severe score was assigned if the milk was grossly abnormal, there was firmness or swelling of the affected mammary gland, and at least 2 of the following systemic disease signs were seen: rectal temperature ≥ 39.5 °C, hydration score showing moderate to marked enophthalmos, and attitude score showing signs of marked depression [19].

All experimental procedures were performed according to the Italian legislation, following approval by the ethics committee of University of Milan.

### *S. aureus* bacterial counts

For determination of *S. aureus* bacterial counts (cfu/mL), series of dilutions (10^2^ to 10^8^) were prepared from 1 mL of each sample of milk, diluted with 9 mL of a 0.1% saline peptone solution. Then 0.1 mL of the serial dilutions were inoculated on the surface of Baird-Parker agar and spread with a spatula. The incubation was done at a temperature of 37 °C for 16 h (overnight incubation).

### Milk fat globule collection

Samples were centrifuged at 2000 × *g* for 10 min at 4 °C to isolate milk fat. The supernatant fat layer was transferred to a new 50 mL-Falcon tube using a sterile spatula. Then, 500 μL of fat were put into a 15 mL-Falcon and 1.5 mL of TRIzol^®^ LS solution (Invitrogen, Life Technologies, Carlsbad, California, USA) was added and the tube was vortexed vigorously prior to storage at -80 °C. The entire process of milk sample collection and storage of MFG was completed within 2 h and all procedures were carried out at 4 °C.

### Tissue collection for laser capture microdissection

At the end of the experimental protocol (30 hours post infection (hpi)), goats were slaughtered, according to surgical and experimental procedures in compliance with the policy of INRA’s Animal Care Committee, after milk sampling. Tissue samples were collected aseptically from the five goats within 10 min after slaughtering. A piece of deep alveolar parenchyma, without visible connective tissue, was removed from the left udder (infected) and right udder (uninfected). The collected tissue was washed in cold PBS solution (on ice), 5 mm^3^ pieces of tissue were cut and embedded into OCT^®^ (TissueTek™) in a cryomold of 1 cm^3^ (Bayer™) and immediately placed on dry ice or in a SnapFrost™ system (Alphelys, Elancourt, France) containing isopentane at -80 °C. Samples were stored at -80 °C until further processing. The time delay between slaughtering and tissue freezing was less than 20 min. Laser Capture Microdissection (LCM) was carried out using the Veritas Microdissection system and software (Arcturus, Life Technologies, St Aubin, France), as previously described [20].

### Total RNA extraction

Total RNA was extracted from mammary tissue samples (pieces of deep alveolar parenchyma referred to as MG) taken at 30 hpi on the left (infected) and the right (uninfected) half-udder, using TRIzol Reagent (Invitrogen, Life Technologies) according to the manufacturer’s instructions. Total RNA was extracted from MFG using TRIzol^®^ LS solution (Invitrogen, Life Technologies) following the original manufacturer’s protocol with slight modifications, essentially as described by Brenaut et al. [16]. Prior to RNA quality control, a DNase treatment was carried out to remove any contaminating genomic DNA according to the manufacturer’s protocol (Qiagen, Courtaboeuf, France).

Total RNA was extracted from captured cells using the PicoPure^®^ RNA Isolation Kit (Arcturus, Life Technologies) according to the manufacturer’s instruction protocol, including on-column RNase-free DNase I treatment (Qiagen). CapSure macrocaps with captured cells were inserted into RNase-free 500 μL microcentrifuge tubes containing 25 μL of extraction buffer (XB). The tubes were inverted to allow the reaction between the buffer and the surface of the cap. RNA were extracted from scraped sections (tissue remaining on the slide after capture) by pipetting 50 μL of XB buffer onto the remaining tissue on the glass slide, which was then gently scraped off and transferred into RNase-free 500 μL microcentrifuge tubes. RNA from cap and section scrapes were eluted with 15 μL and 30 μL of elution buffer (EB), respectively.

### RNA quality control and single strand cDNA synthesis

Purity, concentration and integrity of total RNA intended for microarray analysis and qPCR were assessed using two independent techniques. RNA purity was evaluated by absorbance readings (ratios A260/A230 and A260/A280) using the NanoDrop ND-1000 spectrophotometer (Thermo Fisher Scientific, Villebon-sur-Yvette, France). The fluorimetric method and micro-capillary electrophoresis device developed by Agilent Technologies (Les Ulis, France) was used to determine RNA concentration and quality with RNA 6000 Pico LabChip^®^ Kit in the Agilent Bioanalyzer 2100 system. Quality was evaluated using the RNA Integrity Number (RIN) value, introduced by Schroeder et al. [21]. A PCR test was routinely performed to ensure that RNA samples were free from genomic DNA (primers matching with intron sequences). First-strand cDNA was synthesized from 5–10 ng total RNA primed with oligo(dT)_20_ and random primers (3:1, v/v) using Superscript III reverse transcriptase (Invitrogen, Life Technologies) according to the manufacturer’s instructions. Then, 1 μL RNase H (2 U/μL, Invitrogen, Life Technologies) was added to the reaction mix which was incubated for 20 min at 37 °C to remove RNA from heteroduplex. cDNA, thus obtained, was stored at -20 °C.

### Microarray processing and data analysis

A microarray analysis was performed, in a mono color experimental design, to compare gene expression profiles of MFG at 4 time points of infection (0, 12 h, 18 h and 24 h) on the 5 goats. RNA samples were labelled using T7 RNA polymerase, which simultaneously amplifies target material and incorporates cyanine 3-labelled CTP (Agilent Technologies) according to the manufacturer’s instructions. Briefly, total RNA (120 ng) was first reverse transcribed and then converted to labeled cRNA with Cy3 dyes. Before hybridization, quantity and quality of purified cRNA was assessed with the Agilent 2100 Bioanalyzer.

Since goat microarrays were not available, we used the Sheep Gene Expression Microarray, 8 × 15 K (Agilent Technologies) developed for a closely related species (*Ovis aries*), to profile gene expression. Hybridization was performed according to the manufacturer’s instructions. Briefly, each microarray was hybridized with fluorescent labelled (Cy3) cRNA samples. Three hundred ng of each labelled cRNA were fragmented for 30 min at 60 °C in 25X fragmentation buffer. Then, fragmented and labelled cRNA samples were diluted in 2X GE x Hybridization Buffer Hi-RPM and hybridized onto the 15 K ovine microarray for 17 h at 60 °C in a rotating hybridization oven. After hybridization, microarrays were washed twice with washing buffer 1 at room temperature for 1 min and then with washing buffer 2 at 37 °C for 1 min. Microarrays were scanned on an Agilent scanner G2565BA using the extended dynamic range scan mode. Data were processed (including Lowess normalisation) and extracted with Feature Extraction software version 10.5 (Agilent, Technologies).

Statistical analysis of microarray data was carried out using the commercially available software package GeneSpring^®^ GX v10.0.2 software (Agilent Technologies), using the latest gene annotations available on a web-accessible resource established by SIGENAE [22]. Information about this experiment has been deposited in NCBI Gene Expression Omnibus (GEO) with accession number (GSE39315). All data are “Minimum Information About a Microarray Experiment” (MIAME) compliant. First, we applied a quantile normalization on data, which assumes that the distribution of gene abundance is the same for all samples, and spots that did not meet minimum signal intensity were removed. The resulting signal information was analyzed using repeated measures ANOVA with a Benjamini-Hochberg correction for multiple comparisons. This approach was used since the data correspond to a longitudinal study which measures each individual at each of several time points. We note that a standard ANOVA assumes independence between time points and is not able to differentiate between the variability within each individual (i.e. across time points) and the variability between individuals, resulting in inflated error variance estimates. By accounting for the correlation among repeated measurements for each individual within the model, these error variance estimates can thus be reduced. This analysis included pair wise comparisons between time points (0 h vs. 12 h, 12 h vs. 18 h, and 18 h vs. 24 h), which yielded a list of probes that were differentially expressed in at least one of the pairs considered. A first filter was applied to select the genes that displayed an adjusted *p*-value less than 0.05. The output of this analysis was additionally filtered by fold expression, generating lists of differentially expressed genes with at least 1.5-fold change. This final set of genes was then referenced to biological functions with Ingenuity Pathways Analysis software v6.0 (IPA, Ingenuity Systems, Redwood City, CA, USA). The contribution of genes to the identified function was explained with an associated *p*-value, calculated with a right-tailed Fisher’s exact test.

To identify temporal relationships among this subset of genes, a gene regulatory network was reconstructed from the expression data using an approach specifically developed for replicated longitudinal data, called Empirical Bayes Dynamic Bayesian Networks (EBDBN), implemented in the ebdbNet package in R [23]. Briefly, the EBDBN method makes use of an iterative empirical Bayes estimation procedure for a linear Gaussian state-space model. This approach has the advantage of being able to simultaneously model linear relationships among genes and a set of unobserved hidden variables (e.g., proteins, transcription factors, or other unobserved cellular entities) over time, as well as to explicitly model feedback loops of expression from one time point to another. Using the approach detailed in the package, the dimension of the hidden variables was chosen to be 2. The model was run for 10 different initializations, and gene-to-gene interactions were retained only if identified in at least 9 runs at a significance level of 95%.

### Primer design and real time quantitative PCR

RNA isolated from goat MFG and microdissected MEC were used to determine the expression of selected genes of interest by real time quantitative PCR (qPCR) as described by Bevilacqua et al. [24], using the SYBR green PCR master mix and an ABI Prism 7900 Sequence Detection System (Applied Biosystems, Life Technologies). The reference sequence for primers was taken from a bovine database. Expressed sequence tags (EST) of caprine sequences found by the BLASTn search and reference were aligned to create one consensus. To eliminate the risk of genomic DNA amplification we systematically chose primers that hybridize on exon-exon junctions. Only junctions with great homology between sequences were chosen for primer design. Then primers were designed using Primer Express Software version 2.0 (Applied Biosystems, Life Technologies), and purchased from MWG Biotech (Table 1). First, primer efficiency was validated with a standard curve of four serial dilution points (ranging between 1 ng and 1 pg of reverse transcribed total RNA) and a no template control (NTC). A qPCR amplification mixture (20 μL) contained 5 μL single strand cDNA template, 10 μL 2X Power SYBR Green PCR Master Mix buffer (Applied Biosystems, Life Technologies) and 1.2 μL of forward and reverse primers (5 μM) to reach a final primer concentration of 300 nM. After optimization of qPCR systems (efficiency ranging between -3.30 and -3.45), amplification reactions were run (in triplicate). Tests for non-amplification of genomic DNA was carried out systematically. The results generated by the Sequence Detection Software (Applied Biosystems, Life Technologies, version 2.3) were exported as tab-delimited text files and imported into Microsoft Excel for further analyses. Relative quantification analysis was performed using the software program qBase [25] in which relative expression levels were normalized with respect to the selected reference genes (*RPS24* and *PPIA*), following the “Minimum Information for Publication of Quantitative Real-Time PCR Experiments” (MIQE) guidelines [26].

**Table 1:** Primer sequences used for qPCR experiments

**Genes**	**Primers**	**Sequence 5′ > 3′**	**Amplicon size, nt**
α_s2_-casein (*CSN1S2*)	forward	CTG GTT ATG GTT GGA CTG GAA AA	76
	reverse	AAC ATG CTG GTT GTA TGA AGT AAA GTG	
κ-casein (*CSN3*)	forward	AGG TGC AAT GAT GAA GAG TTT TTT C	66
	reverse	CCC AAA AAT GGC AGG GTT AA	
Cluster of differentiation 3 epsilon (*CD3e*)	forward	ACG CTG TAC CTG AAA GCA AGA	118
	reverse	AAT ACA CCA GCA GCA GCA AG	
Cluster of differentiation 68 (*CD68*)	forward	GAT CTG CTC TCC CTG AAG CTA CA	79
	reverse	CAT TGG GAC AAG AGA AAC TTG GT	
Cluster of differentiation 18 (*CD18*)	forward	AGC GAC CTC AGG GAG TAC CA	65
	reverse	TTA TCG TTG TTC CAC TGG GAC TT	
Chemokine 4 (*CCL4*)	forward	CAG CCG TGG TAT TCC AGA CC	109
	reverse	CTC GGA GCA GCT CAG TTC AGT	
Cyclophilin A (*PPAI*)	forward	TGA CTT CAC ACG CCA TAA TGG T	62
	reverse	CAT CAT CAA ATT TCT CGC CAT AGA	
Galectin 3	forward	GTG GTA AAC CTT TCA AAA TAC AAG TGC	101
	reverse	ATT TTT CAC CCG ATG ATT GTA CTG	
G protein-coupled receptor 97 (*GPR97*)	forward	GAG ATC ACC TTC TCC CAC CAG	204
	reverse	TGT GGA GCA GCC CAA GGA	
Interleukin 8 (*IL-8*)	forward	TGA GAG TGG GCC ACA CTG C	103
	reverse	CAC AAC CTT CTG CAC CCA CTT	
Keratin 14 (*KRT14*)	forward	CCC AGC TCA GCA TGA AAG C	57
	reverse	AGC GGC CTT TGG TCT CTT C	
Serum amyloid A3 (*SAA3*)	forward	CTG GGC TGC TAA AGT GAT CAG TAA C	69
	reverse	CCC TTG AGC AGA GGG TCT GTG ATT	
S100A12	forward	TCC ACC AGT ACT CCA TCC GG	102
	reverse	TGG TGT TTT TGA GGC AGT TGG	
Serglycin	forward	TCC AGC AGA ATC CCA CCT CTA	107
	reverse	CCA GAA CCT GAT CCT GAG ACG	
SerpinA1	forward	AAG AAA TAT GCA AGT TCT GCC AAT T	101
	reverse	ACC CTG TTG ATG CCC AGT TC	
TNFα	forward	CAG AGG GAA GAG CAG TCC CC	101
	reverse	TGG GCT ACC GGC TTG TTA TTT	
Toll-like receptor 2 (TLR2)	forward	TAA ACT TGA GAG TGG AGG TCA AAT CA	101
	reverse	TCA GAG GCT CCT TCC GTG G	
Interleukin 1beta (IL-1β)	forward	GAC AAC AAG ATT CCT GTG GCC	101
	reverse	TCT ACT TCC TCC AGA TGA AGT GT	
Pentraxin 3 (PTX3)	forward	CCG AGC TGT GCA GGG CT	101
	reverse	GCA CGC TTG CAA AAA TCT TCT T	
Cathelicidin	forward	GAG AAT GGG CTG GTG AAA CAG	107
	reverse	GGG CGA AGT CTC CTC ACA CTC	
24S ribosomal protein (*RPS24*)	forward	TGG TGGTGG CAA GAC AAC TG	66
	reverse	TTC TTC GCG TAA TCC AAG GAA	

Each pair of primers amplifies the target cDNA (amplicon sizes ranging between 57 and 248 nucleotides) in its 3′ UTR. Primer pairs were designed with primer Express Software v2.0 (Applied Biosystems) except for 24S ribosomal protein primers, which were manually designed.

### Statistical analysis

Reliability of reference genes (*RPS24* and *PPIA*) was evaluated with the GeNorm Visual Basic application for Microsoft Excel as described by Vandesompele et al. [27]. Statistical analyses were performed using the R software, with the Rcmdr package. The non-parametric Mann–Whitney *U*-test was used to analyse the differences in relative expression for each gene of interest between MG and MFG, non infected versus infected, at 12 h, 18 h and 24 hpi but also between infected MG and microdissected MEC, at 30 hpi.

## Results

### Kinetics of pathological features: an increase of *S. aureus* in milk followed by an increase of somatic cells in milk

**Figure 2:**
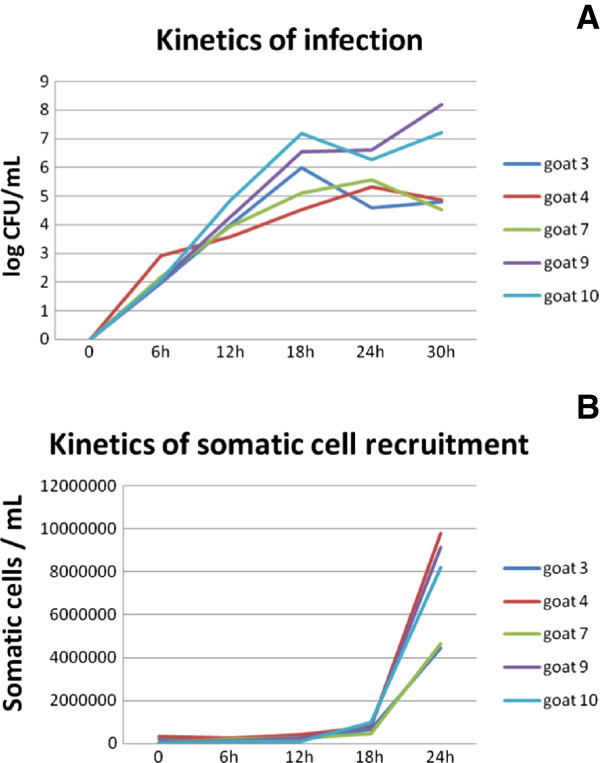
**Kinetics of infection (A) and somatic cell recruitment (B). A**: *Staphylococcus aureus* colony forming unit (CFU/mL) evolution during the 24 first hpi. **B**: Somatic cell counts (SCC/mL) in the left (infected) udder of the 5 goats challenged by *S. aureus*. The control PBS-infused udders remained free from detectable infection throughout the study for the five goats.

Prior to experimental infection, goats were controlled to be free from *S. aureus* and other IMI throughout the three weeks of lactation to avoid the influences of adaptative immune mechanisms. Following administration of 10^3^ CFU/mL of *S. aureus* DV137 to the left udder and the same volume of sterile pyrogen-free PBS into the right udder, left udders contained only the pathogen used for infection whereas the control udder (right) remained bacteriologically negative during the observation period. A high number of pathogens were found 18 h after infection, reaching 4 × 10^6^ CFU/mL and then decreasing to 1.5 × 10^6^ CFU/mL at 24 hpi (Figure 2). Mild mastitis symptoms were concomitant with increased somatic cell counts (SCC) in milk. In infected udders, the number of somatic cells significantly increased from 893.8 × 10^3^ at 18 hpi to 53 × 10^5^ cells/mL at 24 hpi, while SCC in PBS-infused udder halves showed no significant change throughout the period with a mean value of 980 (± 147) × 10^3^ cells/mL (Figure 2). Thus, all infected goats showed a sharp increase of *S. aureus* in milk at 18 hpi which was followed by a significant increase of SCC in milk at 24 hpi. Thirty hours after experimental challenge all goats showed severe signs of clinical mastitis: the milk was grossly abnormal, there was firmness and swelling of the affected left mammary gland and the rectal temperature was ≥ 39.5 °C (40.2 ± 0.5 °C).

### RNA sampling from MEC in their physiological context, during infection

Two different sampling methods were used to access MEC materials from each of the 5 goats throughout the time course of the IMI challenge with *S. aureus* (Figure 1). The first method consisted in milk collection, every six hours post infection, and extracting RNA from cytoplasmic crescents trapped in MFG during the secretion process from MEC. The RNA integrity number (RIN) of total RNA isolated from MFG was estimated, on average, to 7.5 ± 0.5. Then, at the end of this first part of the protocol, mammary tissues were collected on slaughtered goats (*n* = 5) and MEC captured from sections of mammary tissue by Laser Capture Microdissection (LCM). RIN for RNA extracted from microdissected cells were of comparable quality (6.8 ± 0.4) with RNA obtained from MFG. Combining these two techniques is a convenient approach to follow MEC response in a physiological context, provided that contamination by other cell types can be excluded. To assess such an eventuality, qPCR systems designed to quantify specific markers for mammary myoepithelial cells (*Krt14*), lymphocytes (*CD3e*), neutrophils (*CD18*), and macrophages (*CD68* and *CD18*) were implemented [16].

#### RNA extracted from MFG highly reflect MEC gene expression pattern, up to 18 hpi

**Figure 3:**
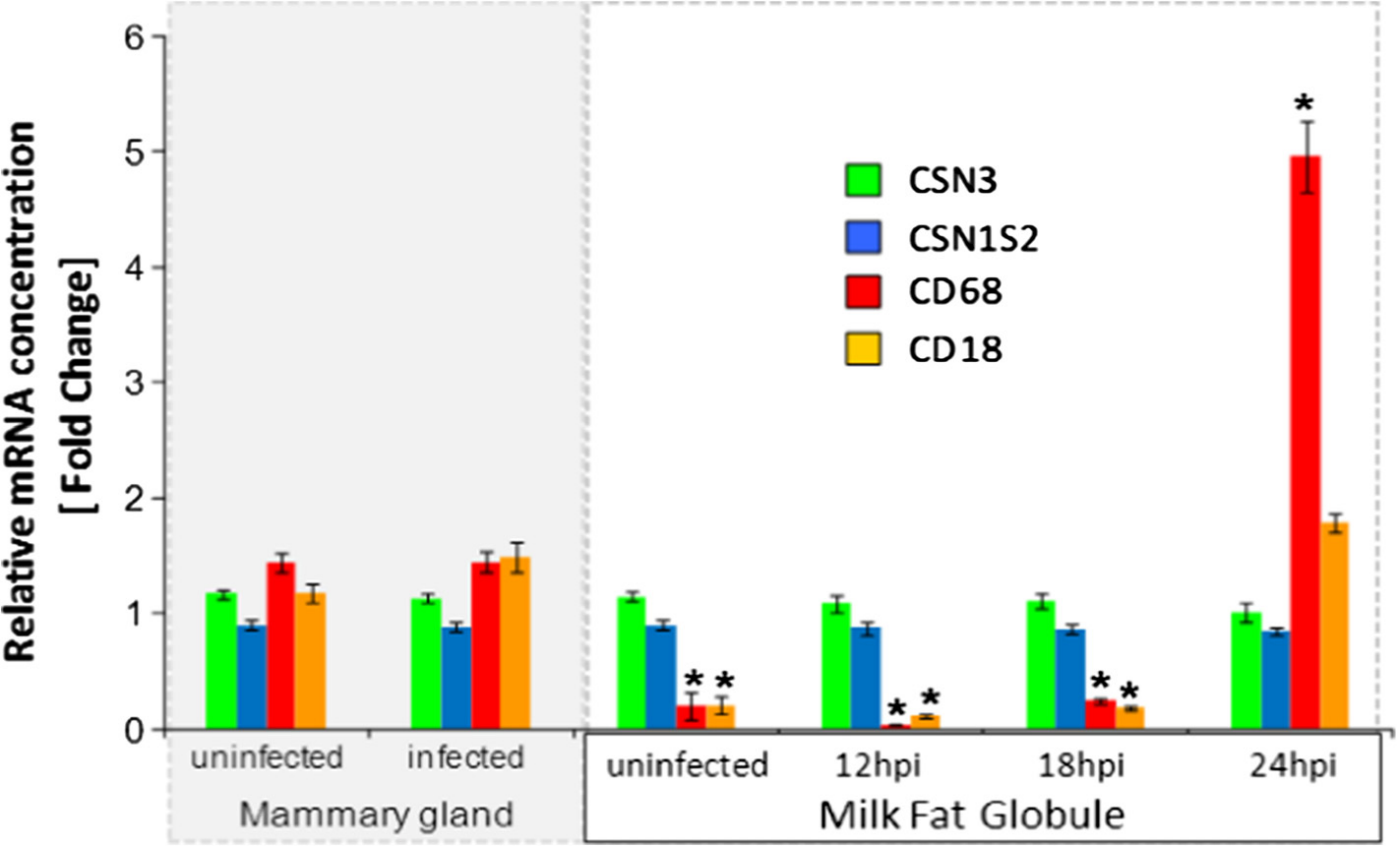
**Determination of specific markers by qPCR to assess the level of contamination of RNA extracted from milk fat globules by RNA from immune cells.** Relative expression (± SEM) is given for specific gene markers for MEC *CSN3* (κ-casein, green), *CSN1S2* (α_s2_-casein, blue), macrophages (*CD68*, red) and macrophages + neutrophils (*CD18*, orange) in the left half-udder (infected) or the right half-udder (uninfected), sampled at 30 hpi on slaughtered goats (mammary gland, left), and in milk fat globules (right), before infection (uninfected) and after IMI challenge with *S. aureus* (12 hpi, 18 hpi and 24 hpi). *Significance is relative to fold change in expression of uninfected MG (adjusted *p*-value < 0.05).

During MFG preparation, some immune cells (mainly neutrophils and to a lower extent macrophages) present in milk can be trapped in the cream layer. To evaluate the level of RNA contamination, qPCR analyses were carried out using specific gene markers of these immune cells: *CD68* for macrophages and *CD18* for neutrophils and macrophages. Two markers of MEC (*CSN3, CSN1S2*) were also included as positive controls which were expressed at the same level in MFG of uninfected and infected udder, at all time points post infection (Figure 3). Regarding the level of contamination by macrophages and neutrophils, qPCR analyses showed that these immune cells were actually present but in very low amounts, between 7 to 9-folds lower in MFG than in the whole deep mammary alveolar parenchyma (MG), until 18 hpi (Figure 3). These very low values (RQ mean *CD68* = 0.15 ± 0.05 and RQ mean *CD18* = 0.17 ± 0.03, between T0 and T18) reflect a slight contamination by macrophages and neutrophils, during MFG preparation. Contamination increased and became non negligible, essentially for macrophages, with an RQ mean for *CD68* reaching 4.96 ± 0.31, at 24 hpi, whereas it was 30-folds lower at 18 hpi. Therefore, at least until 18 hpi, responses to IMI challenge observed via MFG transcriptome analysis have to be essentially attributed to MEC, whereas from 24 hpi and beyond, a part of the gene expression data is influenced by infiltrating immune cells trapped in the cream layer during MFG preparation.

#### Selectivity of MEC capture by LCM on cryo-sections of mammary tissue

**Figure 4:**
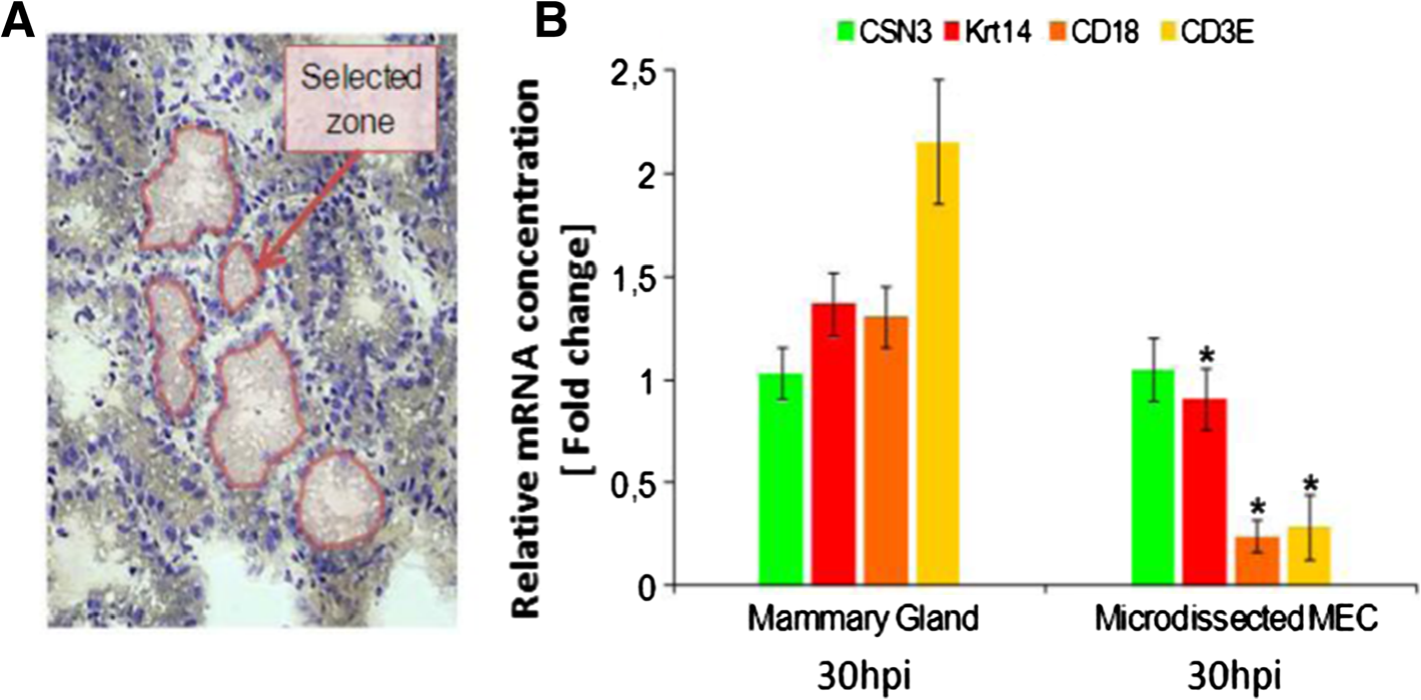
**Selectivity of MEC capture by Microdissection from infected mammary tissue, assessed by real-time qPCR. A)** Section of infected mammary tissue with the zone selected for laser capture microdissection (pink). **B)** Relative expression (± SEM) of specific marker for MEC (*CSN3*, green bar) and for putative contaminating myoepithelial cells (*Krt14*, red bar), macrophages and neutrophils (*CD18*, orange bar) and lymphocytes (*CD3e*, yellow bar) in infected mammary gland (left) and in microdissected infected MEC (right), at 30 hpi*.* *Significance is relative to fold change in expression of infected MG (adjusted *p*-value < 0.05).

To provide additional insight into the immune role associated with MEC during infection, we investigated gene expression profiles of microdissected MEC. Indeed, 24 hpi, MFG extracted material was no longer representative of MEC owing to a significant contamination of MFG by immune cells (see above). Therefore, at the end of the experimental milking sampling protocol (i.e. 30 hpi), mammary tissue was collected on slaughtered goats in order to capture MEC from tissue cryo-sections, using LCM (Figure 1). This technique makes it possible to capture MEC while they are in their physiological context and therefore to obtain RNA specifically from this cell type at 30 hpi, thus allowing a comparison with RNA isolated from MFG at the same stage. However, infection leads to an infiltration of immune cells within the secretory parenchyma which affects tissue morphology and reduces the surface on which LCM could be carried out (Figure 4B). Consequently, only four goats were sampled given the morphology which was strongly affected by infection and therefore not easily suitable for microdissection. To assess the selectivity of MEC capture, mRNA transcript level of a MEC specific marker (*CSN3*) was estimated by measuring the relative expression between captured cells and their corresponding mammary tissue scrapes. The amount of mRNA encoding CSN3 was unchanged (RQ mean = 1.02). Furthermore, *CD18* and *CD3e* expression decreased dramatically (6 to 10-folds reduction) in captured cells as compared with the whole mammary tissue, thus suggesting a negligible presence of immune cells in laser-captured cells, further demonstrating the efficiency of LCM (Figure 4B). However, a weak expression was systematically recorded for *Krt14*, in microdissected MEC, reflecting a slight contamination by mammary myoepithelial cells (MMC) during the course of the capture process, since it is known that *Krt14* is expressed by MMC only [20]. This is probably due to the very close proximity between MMC and MEC and to the difficulty of disassociating luminal MEC from MMC bordering the basal lamina that separates the epithelial layer from the extracellular matrix.

### MEC response during the first 24 h following IMI challenge with *S. aureus*

Gene expression profiling of MFG at the early steps (between 0 and 24 h) of the experimental IMI challenge with *S. aureus* was examined for the 5 goats of our experimental design, using microarray technology to identify a repertoire of differentially expressed genes in goat MEC in response to infection. Microarray results showed that the most highly expressed genes, at the 4 time points of infection (including 24 hpi), were the signature of lactating MEC gene expression. Indeed, genes coding for milk proteins (*LALBA, CSNs* and *BLG*) and ribosomal proteins involved in protein synthesis, RPL and RPS, gave strong signals. Likewise, amongst these highly expressed genes, we also found genes involved in synthesis and secretion of \fatty acids and triglycerides (*FASN, XDH, ADRP, SCD* and *DGAT1*), as well as the gene encoding UDP-galactosyltransferase (*B4GALT1*) which participates in glycoprotein post-translational modification and lactose synthesis. Furthermore, microarray data are consistent with the results of qPCR analyses performed to assess the level of contamination of MFG by immune cells. Indeed, specific macrophage and neutrophil gene markers present on the sheep microarray indicate an absence of immune cells up to 18 hpi whereas the contamination by neutrophils and macrophages began to be noticeable at 24 hpi. Consequently, only the first three conditions (T0, T12 and T18) were considered in the differential analyses done to highlight MEC immune response to IMI. Globally, repeated measures ANOVA analysis of microarray data revealed that a limited number of genes were impacted in their expression at the early stages of infection. Among the 15 208 probe set present on the Sheep Gene Expression Microarray (8 × 15 K), a total of 47 probes (39 annotated genes) were significantly differentially expressed in response to IMI challenge after a Benjamini-Hochberg correction [28] to control the false discovery rate (adjusted *p*-value inferior to 0.05). These genes (Table 2) showed at least a 1.5-fold difference in expression in one of the pair wise comparisons (0 vs. 12 h, 12 vs. 18 h). Among these 39 differentially expressed genes, only 12 genes are differentially expressed at 12 hpi, while all 39 are differentially expressed between 12 and 18 hpi. This limited but increasing number of differentially expressed genes was consistent with the fact that gene expression analyses were performed at the very early stages of infection. Regarding the 0 h vs. 12 h comparison, fold changes were relatively low (ranging between 1.5 and 2.7) with a majority of genes (11) down-regulated. Only 3 genes were up-regulated corresponding to a chemokine (CCL-2), the immediate early response 3 protein (IER3) and the phosphodiesterase 4B cAMP-specific (PDE4B). Between 12 and 18 hpi, there were more differentially expressed genes, and the balance between up and down regulated genes was reversed. Only 3 genes were down-regulated during this period with low fold changes (1.6 to 1.7). In contrast, 36 genes were up-regulated, with fold changes ranging between 1.5 and 21 (Table 2). Genes whose expression was highly increased, were mainly involved in the inflammatory response [interleukin-1 receptor (*IL-1RN*), S100 calcium-binding protein A12 (*S100A12*), Interleukin-1β (*IL-1β*)], in chemotaxis [interleukin 8 (*IL-8*), chemokine C-C motif ligand 4 (*CCL4*)], in cell adhesion [tumor necrosis factor alpha-induced protein 6 (*TNFAIP6*), platelet/endothelial cell adhesion molecule (*PECAM1*)], in opsonization by enhancing phagocytic activity of infiltrating immune cells [pentraxin 3 (*PTX3*)] and in apoptosis [serglycin (*SRGN*), Bcl-2 related protein A1 (*BCL2A1*)].

**Table 2:** **The 39 differentially expressed genes (adjusted *p*-value < 0.05 and fold change > 1.5) in Milk Fat Globules of goats (*n*** **= 5), in response to an experimental IMI with *S. aureus, *** **in at least one of the two pairwise comparisons (T0 vs. 12 hpi; 12 hpi vs. 18 hpi)**

**Description**	**Gene symbol**	**Biological function**	**Fold change T0/T12 (sens of regulation)**	**Fold change T12/T18 (sens of regulation)**
Arachidonate 5-lipoxygenase-activating protein	*ALOX5AP*	Leukotriene metabolic process	*1.1*	1.7 (up)
**Bcl-2-related protein A1**	***BCL2A1***	**Apoptosis**	***1.0***	**5.1 (up)**
Chemokine (C-C motif) ligand 2	*CCL2*	Inflammatory response, chemotaxis	1.6 (up)	1.6 (up)
**Chemokine (C-C motif) ligand 4**	***CCL4***	**Inflammatory response, chemotaxis**	***1.1***	**6.8 (up)**
**C-type lectin domain family 4 member E**	***CLEC4E***	**Immune response**	***1.3***	**6.1 (up)**
Cathepsin Z	*CTSZ*	Proteolysis	*1.4*	1.5 (up)
Early growth response protein 1	*EGR1*	Regulation of transcription, cell proliferation	*1.1*	1.6 (up)
Early growth response 3	*EGR3*	Apoptosis, chemotaxis	*1.1*	2.3 (up)
Fc fragment of IgG, low affinity IIIa, receptor	*FCGR3A*	Immune response	1.7 (down)	1.8 (up)
Proto-oncogene protein c-fos	*FOS*	Regulation of transcription, cell proliferation	*1.1*	2.5 (up)
Growth arrest and DNA-damage-inducible protein GADD45 alpha	*GADD45α*	Cell cycle	1.8 (down)	3.2 (up)
Immediate early response 3	*IER3*	Inflammatory response	1.5 (up)	2.7 (up)
Interleukin-18-binding protein Precursor	*IL18BP*	Regulation of transcription, cell proliferation	*1.3*	1.7 (down)
**Interleukin 1 beta**	***IL-1β***	**Inflammatory response, cytokine biosynthesis**	**1.5 (down)**	**5.6 (up)**
**Interleukin-1 receptor**	***IL1RN***	**Inflammatory response**	***1.0***	**9.8 (up)**
Interleukin-2 receptor gamma chain	*IL2RG*	Regulation of gene expression, alpha-beta Regulatory T cell differentiation	*1.2*	1.5 (up)
**Interleukin 8**	***IL-8***	**Chemotaxis**	***1.3***	**8.5 (up)**
Metallothionein-1A	*MT-1A*	Cellular response to zinc and cadmium ion	*1.4*	1.7 (down)
Phosphodiesterase 4B, cAMP-specific	*PDE4B*	Signal transduction	2.1 (up)	2.1 (up)
Platelet/endothelial cell adhesion molecule	*PECAM1*	Cell adhesion	*1.4*	3.0 (up)
**Pentraxin-related protein**	***PTX3***	**Regulation of phagocytosis**	***1.2***	**4.8 (up)**
Regulator of G-protein signaling 2	*RGS2*	Cell cycle	*1.2*	2.2 (up)
Ras-related associated with diabetes	*RRAD*	Protein transport	*1.0*	1.6 (up)
**S100 calcium-binding protein A12**	***S100A12***	**Inflammatory response**	**1.9 (down)**	**8.0 (up)**
Protein strawberry notch homolog 2	*SBNO2*	Regulation of transcription, cell proliferation	*1.4*	1.6 (down)
Serine dehydratase	*SDS*	Cellular amino acid metabolic process	*1.3*	5.7 (up)
Alpha-1-antitrypsin	*SERPINA1*	Inflammatory response, acute phase response	*1.3*	2.1 (up)
**Solute carrier family 2, facilitated glucose transporter member 3**	***SLC2A3***	**Carbohydrate transmembrane transport**	***1.4***	**6.9 (up)**
Suppressor of cytokine signaling 3	*SOCS3*	Inflammatory response	*1.4*	2.4 (up)
**Spleen focus forming virus (SFFV) proviral integration oncogene**	***SPI1***	**Regulation of transcription, cell proliferation**	**2.7 (down)**	**4.4 (up)**
**Serglycin**	***SRGN***	**Apoptosis**	***1.3***	**8.4 (up)**
Tumor necrosis factor alpha	*TNFα*	Inflammatory response, anti aptoptosis	*1.3*	2.9 (up)
**Tumor necrosis factor alpha-induced protein 6**	***TNFAIP6***	**Cell adhesion**	***1.0***	**9.1 (up)**
Tristetraprolin	*TTP*	Inflammatory response	*1.0*	1.6 (up)
Thioredoxin-interacting protein	*TXNIP*	Response to oxidative stress	2.1 (down)	1.5 (up)
021030OOCX008067HT OOCX Ovisaries cDNA			1,7 (up)	4.0 (up)
MGC165862 protein			1.6 (down)	3.4 (up)
**020605OCS411016083HT OCS4 Ovisaries cDNA**			***1.2***	**21.0 (up)**
Taste receptor type 2 – Bos taurus (Bovine), partial (26%) [TC17737]			1.5 (down)	2.7 (up)

Adjusted p-value < 0.05 and fold change > 1.5 are in bold, in at least one of the two pairwise comparisons.

#### Gene network analysis of differentially-expressed gene lists

**Figure 5:**
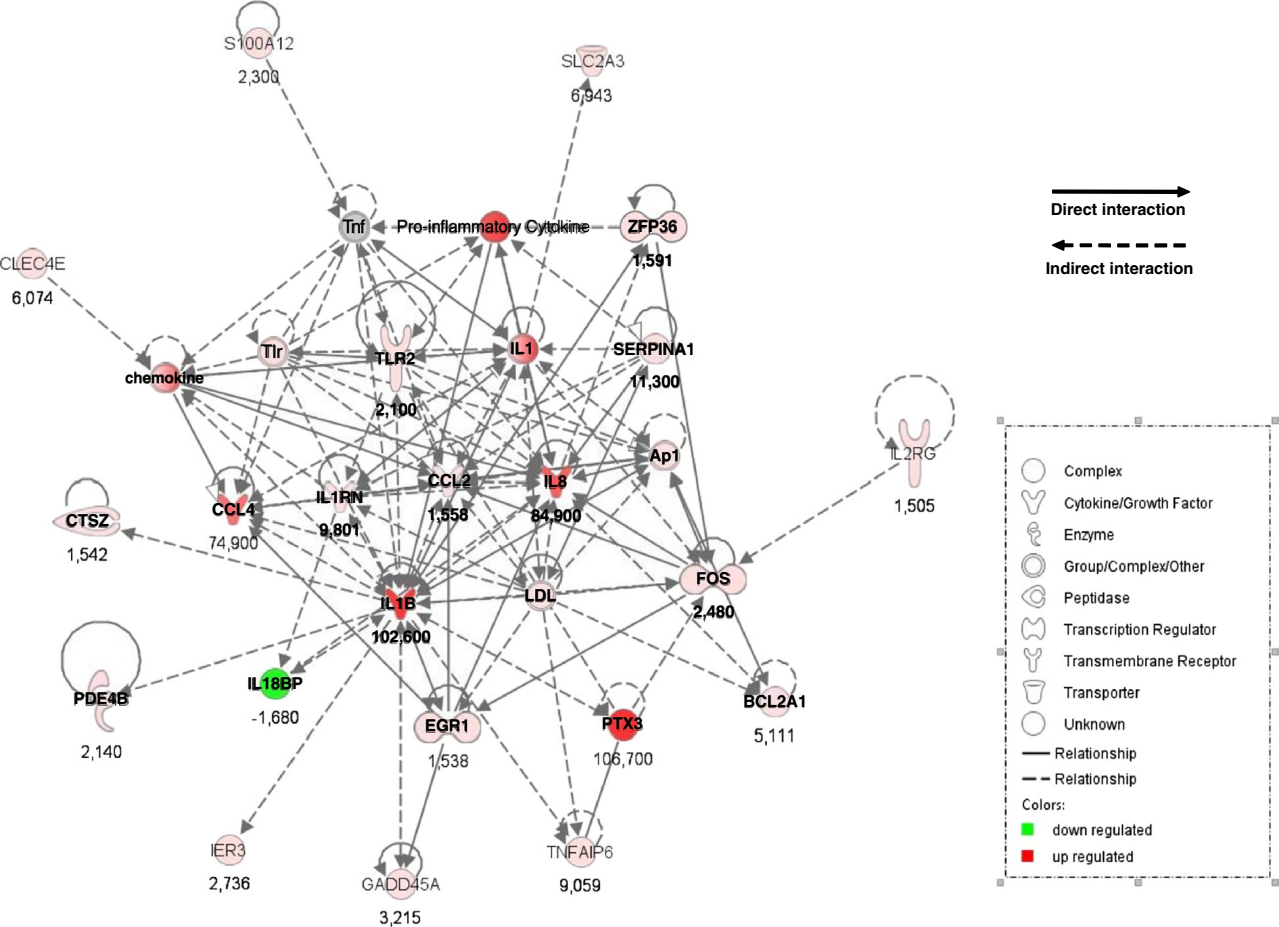
**Ingenuity Pathway Analysis (IPA) of the 39 differentially expressed genes in MFG transcriptome at 18 hpi vs*. *before infection (T0).** The IPA legend defining the symbols depicted in IPA networks is given in the inset. Direct interaction is in solid line whereas indirect interaction is indicated by a dotted line.

Systemic identification and grouping of differentially-expressed genes into biological networks was performed using the software packages Ingenuity Pathway Analysis. Analysis of gene interactions and pathways was performed using the differentially-expressed gene sets (0–12 h and 12–18 h) after staphylococcal stimulation, independently. We observed that top networks obtained at 12 hpi deal with cellular movement with 12 molecules on 14 molecules annotated taking part in this network. At 18 hpi, the most relevant biological networks were related to inflammatory response and infectious disease with 27 molecules on 32 molecules annotated (Figure 5). Moreover, for each comparison, inflammatory response and cell to cell signalling and interaction were among the top biological functions that came up with highly significant *p*-values (1.09 × 10^–14^-8.77 × 10^–05^ and 1.09 × 10^–14^-9.69 × 10^–05^, at 12 hpi, respectively and 1.55 × 10^–20^-8.04 × 10^–04^ and 1.65 × 10^–14^-7.43 × 10^–04^ at 18 hpi, respectively.

**Figure 6:**
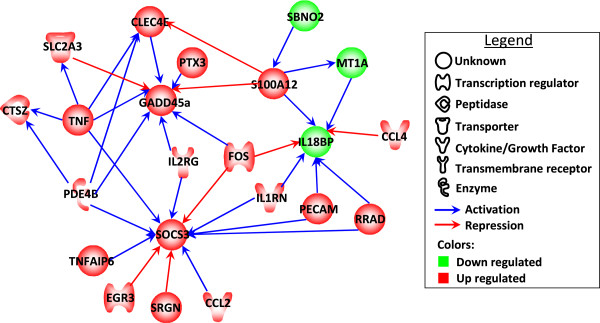
**Interaction network analysis of the differentially expressed genes at the early stages of infection.** This consensus network represents the relationships identified by the analysis with the EBDBN approach (see Materials and methods).

Using the Empirical Bayes Dynamic Bayesian Network (EBDBN) algorithm in order to identify temporal relationships among the 39 differentially expressed genes, we were able to construct an interaction gene network. Using this approach, it appears that *GADD45α*, *IL18BP* and *SOCS3* are highly regulated genes in the network with a high degree of outward connectivity to other genes that are for the most up-regulated (Figure 6). *TNFα*, *PDE4B* and *S100A12* are conversely key genes encoding molecules that activate or repress the expression of other genes of the network.

#### Confirmation of differentially expressed genes by qPCR

**Figure 7:**
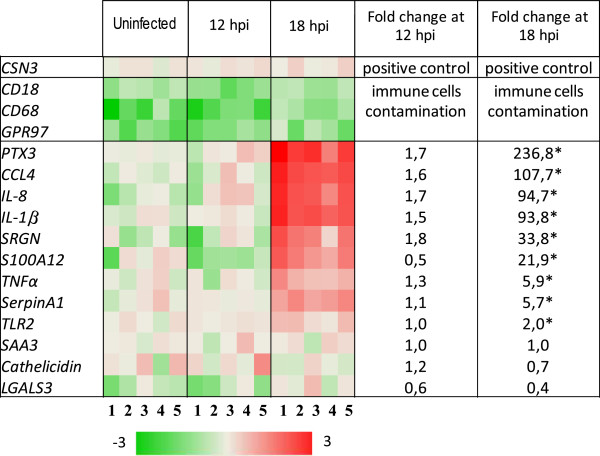
**Heat map of up- and down-regulated genes related to innate defense, selected from MFG transcriptome analysis of the IMI time course challenge with *S. aureus*.** Gene expression was assessed using qPCR; genes shown in red are up-regulated and those shown in green are down-regulated in MFG infected at 12 hpi and 18 hpi, relative to MFG from the uninfected half-udder. Data are expressed in log_10_ ratios with respect to the reference genes (*RPS24* and *PPIA*), at each time point and represent the five biological replicates at each time point.

**Figure 8:**
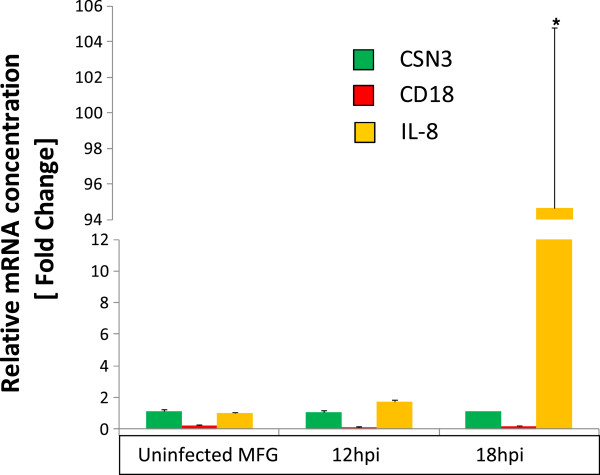
**Expression of *IL-8 *in MFG, at different time points of infection (12 hpi and 18 hpi) induced by an *S. aureus *challenge.** Abundance of mRNA arising from the gene encoding the pro-inflammatory cytokine IL-8 (yellow bar) is expressed relative (± SEM) to transcripts from *CSN3*, a specific MEC marker (green bar) and from *CD18*, a specific marker for macrophages and neutrophils (red bar). *Significance is relative to the fold change in expression taking MFG from milk of the right uninfected half-udder as reference (adjusted *p*-value < 0.05).

A total of 16 genes were chosen to be confirmed by qPCR and to substantiate the involvement of the key biological pathways identified. Together with the positive marker (*CSN3*) and contamination markers (*CD18*, *CD68* and *GPR97*), we chose eight up-regulated genes encoding: pentraxin 3 (*PTX3*), interleukins 1β and 8 (*IL-1β*, *IL-8*), chemokine 4 (*CCL4*), serglycin (*SRGN*), tumor necrosis factor alpha (*TNFα*), SERPINA1 (*SERPINA1*), *S100A12* and four apparently non regulated genes encoding proteins known to be involved in the innate immune response: Toll-like receptor 2 (*TLR2)*, cathelicidin 3 (*CATHL3*), galectin3 (*LGALS3*) and the acute phase serum amyloid A3 (*SAA3*). All of these genes, with the exception of *TLR2*, that were found differentially expressed or not, as defined by the initial microarray screening, conserved the same status when analyzed by qPCR (*P* < 0.05). The reasons why *TLR2* was not identified in the microarray analysis whereas it unambiguously showed a differential expression when measured by qPCR (Figure 7) are not clear. This might be due to a weak specificity of the microarray probe, rather than to the use of a heterologous system since we did not find any difference between goat and sheep *TLR2* mRNA nucleotide sequences that match perfectly. For the other target genes, our results show the same trend (Figure 7) and correlate with microarray data, although systematically and significantly higher in qPCR. For instance, the expression of the gene encoding the pro-inflammatory cytokine IL-8 increased sharply between 0 and 18 hpi (mean fold change = 94.7, with an important variability between goats) whereas the specific MEC marker was constant and the contamination by infiltrating immune cells remained negligible (Figure 8). Indeed, we also observed a sharp increase in the expression of different innate immune genes, such as *PTX3*, *IL-1β*, *CCL4* and *S100A12,* at 18 hpi. Regarding the gene encoding the SAA3 acute phase protein, its expression was unchanged until 18 hpi whereas the expression of *TLR2* showed a slight increase (fold change = 2.0).

Taken together, these results strongly support the notion that during the early stages post-inoculation (before 24 hpi), MEC initiate a prompt response to infection and a sharp induction of innate immune genes that are predominantly associated with the pro-inflammatory response.

### Genes associated with the innate immune response are still expressed in MEC at 30 hpi

Given that the MEC response to infection cannot be analyzed using RNA extracted from MFG at 24 hpi and beyond, due to MFG contamination by immune cells after the burst of SCC in milk, mammary tissue samples collected at slaughtering on goats at 30 hpi were used to monitor MEC contribution using the same qPCR approach on RNA extracted from microdissected MEC. LCM is considered the gold standard method to analyze single cell-type gene expression, since it allows an isolation, under morphological control, of cells of interest (here MEC) in their physiological environment. However, given the limited amount of material extracted, we only measured the expression of a few genes found up-regulated in qPCR at 18 hpi (see above). Thirty hours after the IMI challenge, all the genes analyzed displayed a higher expression than in uninfected MEC (Table 3). The expression of *IL-8* and *PTX3*, for example, increased until reaching a fold change (322 ± 6 and 124 ± 5, respectively) of the same order as that found within the mammary parenchyma (418 ± 9 and 158 ±5, respectively), strongly supporting the notion that IL-8 and PTX3 are synthesized by MEC. This conclusion also applied to SAA3, SERPINA1, and TLR2, thus suggesting an important contribution of this cell-type in initiating the inflammatory response. On the contrary, several genes found up-regulated at 18 hpi using MFG RNA, such as *IL-1β*, *CCL4, TNFα* and *S100A12,* showed a lower expression at 30 hpi as compared with the mammary parenchyma (Table 3).

**Table 3:** Changes in expression of some innate immune genes in the mammary gland parenchyma (MG) and in microdissected MEC, relative to non-infected tissue and non-infected microdissected MEC at 30 hpi, respectively

**Target genes**	**Fold change in MG at 30 hpi**	**Fold change in microdissected MEC at 30 hpi**
*SAA3*	2.71 ± 0.75	3.01 ± 0.70
*SERPINA1*	10.40 ± 1.02	9.56 ± 0.89
*TLR2*	3.68 ± 0.87	2.97 ± 0.53
*PTX3*	158.07 ± 5.20	123.83 ± 5.11
*IL-8*	417.87 ± 8.78	321.85 ± 6.55
*TNFα*	29.57 ± 1.77	16.77 ± 1.21
*CCL4*	8.30 ± 1.26	3.17 ± 0.36
*S100A12*	42.41 ± 1.02	14.92 ± 0.34
*IL-1β*	32.32 ± 2.41	7.55 ± 0.57

**Figure 9:**
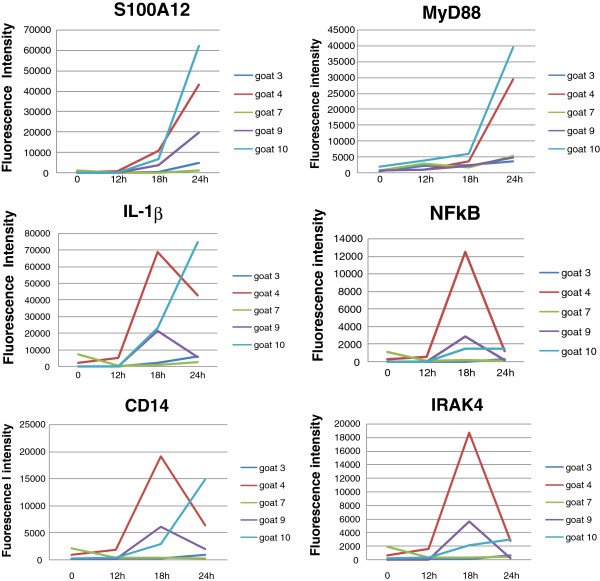
**Individual time-courses expression of selected genes assessed with RNA from MFG using microarrays, during the course of the IMI challenge by *S. aureus*.** Selected genes are the following: *S100A12*, *Il-1β*, *CD14, NFκB, IRAK4* and *MyD88*. Gene expression reflecting abundance of mRNA is given as fluorescence intensity (normalized signal).

This under-expression at 30 hpi could be due either to a specific degradation of the cognate transcripts during the microdissection process or most likely to an evolution in the gene expression patterns. The decreased expression recorded for *IL-1β* and *TNFα* at 24 hpi with the microarray analysis was consistent with this hypothesis, at least for 2 of the 5 goats (Figure 9). On the contrary, dynamics of the expression observed for *S100A12* (Figure 9) and *CCL4* (not shown), between 0 and 24 hpi, showed a pronounced tendency to increase, which seems consistent with the type of effectors and their functions. Since *S100A12* is also expressed by neutrophils and macrophages, this result is very likely due to the presence of leukocytes infiltrating the mammary tissue (mainly neutrophils) in the infected MG samples taken at 30 hpi. Interestingly, it should be mentioned that the gene encoding CD14 antigen, a cell surface protein known to play a pivotal role in mediating recognition of bacterial cell wall components [6,12], followed the same dynamic profile as that of *TNFα* in all five goats. When infection is well established, cellular factors recruited from the blood stream, the expression by MEC of pro-inflammatory cytokines and chemokines declined in favour of the expression of several acute phase proteins.

## Discussion

It has proved to be difficult to reliably analyse in vivo the time course of the pathogen-specific immune response in the udder [29]. Several groups have designed and standardized experimental conditions to reproducibly infect lactating udders from cows [6,30,31] and ewes [32] to generate meaningful results describing molecular mechanisms of host-pathogen interactions. However, infection of animals suffers from among-individual variation, especially regarding dairy ruminant species. In addition, the udder is a complex organ made of several cell types contributing differentially to its immune response [33]. Therefore, to characterize the MEC response, relevant model cells such as primary bovine MEC have been proposed for the molecular analysis of immune defense mechanisms in the udder [12,34,35]. Their potential to express genes of immune defense in response to stimulation with heat-killed bacteria was recently characterized showing pbMEC is capable of secreting large amounts of mediators of inflammatory response, namely IL-8 and TNFα in a pathogen dose dependent fashion [35]. Comparing the inflammation dependent regulation of factors contributing to the complement system between the udder and MEC to those from fully inflamed udders after infection with live *E. coli* pathogens also underlines some regulatory limitations of the pbMEC cell model, which does not properly reflect the mastitis induced regulation of chemokines and the complement system in the udder [14].

The present study is, to our knowledge, the first report describing the in vivo transcriptional host response of MEC in dairy ruminants, early after an IMI challenge with *S. aureus*. We assessed the contribution of MEC to initiate a pro-inflammatory response and to participate in the recruitment of neutrophils and macrophages at the beginning of staphylococcal infection. Usually, in vitro models are used to get an inside view of MEC function, but it remains a simplified and artificial model. A surrogate and less reductionist approach than the in vitro cellular and invasive biopsy methods is the use of “cytoplasmic crescents” enclosed in MFG, first proposed by Maningat et al. [36] in humans and recently validated in goats [16]. However, RNA from MFG, which in theory only contain RNA from MEC, could be contaminated by RNA from infiltrating immune cells, whose population increases in milk during the infection process, and which can be trapped in the cream layer along MFG isolation. Our results show that up to 18 hpi, RNA extracted from MFG is uncontaminated by RNA from immune cells (Figure 3). The expression level of neutrophil and macrophage specific markers was 17 times less than in a non infected mammary gland whose immune cell population is not more than 5% in the cow mammary tissue [37]. Conversely, at 24 hpi, data should be interpreted with care, and we considered it wiser not to treat MFG as fully representative of MEC due to the level of contamination of MFG by immune cells which can then not be negligible. RNA extracted from MFG reflects the specific physiological metabolic process in functional epithelial cells within the mammary gland, only until 18 hpi. However, regarding some genes known to be specifically and highly expressed in MEC, such as genes encoding milk proteins, RNA extracted from MFG remains representative of this cell type. As shown by qPCR experiments, LCM allows obtaining MEC enriched material with low contamination by MMC. In such a manner we were able to complete until 30 hpi our study of MEC response in their physiological context, even though this contamination was slightly higher than that previously reported by Bevilacqua et al. [20] on healthy tissue. This is probably due to tissue damage associated with inflammation leading to a loss of the secretory epithelium cohesion (Figure 4A). However, since MMC were not shown to be able to induce the production of chemoattractants for neutrophils [38], we can assume that this slight contamination of MEC by MMC has in fact little impact on changes in the expression profile induced by the infection and therefore does not interfere with the contribution of MEC in innate immune gene expression.

In addition, throughout the time course of the challenge we confirmed that mRNA molecules encoding proteins involved in the primary function of the udder, i.e. milk synthesis, were not affected by the experimental infection, thus demonstrating that there is no significant loss of function in the mammary tissue, at least during the first 24 h. Therefore, the experimental model measured changes in immune gene responses that are likely to be appropriate to study the initial stages of infection. On the contrary, it was clearly demonstrated in cows that signals are transmitted to the surrounding tissue and to the neighbouring uninfected quarters, very early (within the first 24 h) after contact with *E. coli*[39].

Transcriptome analyses provided here, even though performed with an incomplete repertoire of gene probes, revealed that gene expression profiles clearly changed at 18 hpi with a low number of differentially regulated genes (*n* = 39), concomitantly with a 5 × 10^6^-fold increase of *S. aureus* bacterial count in milk from infected udders. If similar changes occurred in response to the bacterial infection in the neighbouring uninfected udder, as reported by Jensen et al. [40] in bovine, then such changes may have masked some of the differences between the infected and uninfected udder and this could be a reason why the list of differentially expressed genes is relatively short.

The rather late change in gene expression, only 18 hpi, could be explained by the delay to reach sufficient bacterial concentrations in milk. Sensing the presence of bacteria is an important component of innate immunity [5]. Indeed, kinetic studies of experimental clinical mastitis induced by the intra-mammary infusion of *S. aureus* in ovines showed that the inflammatory response (leucocyte reaction) is not initiated until bacterial concentrations reach 4.10^4^ bacteria/mL of milk [41]. Differentially expressed genes, identified in the microarray screening, of which 15 were confirmed by qPCR, indicated that MEC respond to an IMI challenge with *S. aureus* by substantial increase in abundance of mRNA coding for a range of pro-inflammatory cytokines, chemotactic proteins, antimicrobial factors and acute phase proteins. Our results clearly demonstrate that MEC, which form the first line of defense against invading pathogens, are actually able to induce an up-regulation of immune-associated genes involved in the inflammatory response, thus substantiating previous in vitro studies [9,10,12,13,42]. We note that, as pointed out by Gunther et al. [14] in a comparison of primary culture of bovine MEC and udder, such a MEC model, though useful, is generally not considered to reflect what actually happens in vivo. It seems, however, that MFG provides more accurate information to analyze the MEC response, at least during the very first steps of an IMI challenge.

Furthermore, some of the genes found up-regulated in MEC play a key role in orchestrating the regulation of other immune effectors. For instance, the temporal regulatory network, built from the expression data using the Empirical Bayes Dynamic Bayesian Network (EBDBN) algorithm, underlines the first role of MEC in the immune response. This role consists of the recruitment of neutrophils through the up-regulation of *PDE4B* and *TNFα* and in the differentiation and proliferation of lymphocyte T through the up-regulation of *GADD45α*[43]. More precisely, it was observed that the gene encoding the phosphodiesterase PDE4B, which is expressed in the bovine mammary gland [44], plays a key role in immune cell recruitment. Ariga et al. [45] showed that neutrophil recruitment to the lung is impaired in PDE4-deficient mice, by inactivating the second messenger, the cyclic adenosine monophosphate (cAMP), thus abrogating its negative effects on neutrophil recruitment and activation. Jin et al. [46] provide evidence that the gene encoding PDE4B, which is expressed in mouse peritoneal macrophages, is involved in the control of TLR signaling, significantly reducing *TNFα* expression.

The multifunctional pro-inflammatory cytokine TNFα was shown to induce the expression and release of IL-8 by bovine MEC [47]. Our results which are consistent with this, show that genes encoding TNFα and IL-1β and, to a less extent, IL-6 which are typical pro-inflammatory mediators produced in response to TLR stimulation, are up-regulated at 18 hpi. IL-8, a potent chemokine capable of initiating an acute inflammatory response, plays a primary role in the recruitment of neutrophils into the gland [48]. Thus, upon recognizing the pathogen, MEC are able to send out rapidly a strong signal to recruit cellular factors of immune defense (macrophages, neutrophils) from the blood stream into the infected mammary gland. Indeed, this change in gene expression was then followed, 24 hpi, by a massive arrival of cells in milk (Figure 2B), mainly represented by neutrophils [49].

Our results also suggest that the gene suppressor of cytokine signalling 3 (*SOCS3*) occupies a crucial position in the network arising from the temporal analysis. This gene, important for the mammary tissue homeostasis [50,51], encodes an intracellular inhibitor of cytokine signaling that acts in a classical negative feedback loop [52,53]. This is consistent with the fact that the up-regulation of different cytokine encoding genes such as *IL-1β*, *TNFα* and *CCL4* by MEC is restricted at the very early stages of infection, before a decrease at 30 hpi. This short-lived up-regulation was also observed during in vitro studies [15]. Nevertheless, in comparison with in vitro studies, after challenging primary bovine MEC cultures (pbMEC) with heat-inactivated preparation of *S. aureus*, the induction of cytokine-encoding genes such as *IL-1β* and *IL-8* occurred later on in our study. Gunther et al. [35] also observed the same results with a stronger and earlier increase in mRNA abundance in pbMEC challenged with *E. coli* than that found in the udder as caused by acute mastitis. This ability to respond more quickly and strongly could be due to the fact that MEC in culture does not properly reflect the mastitis induced-regulation of cytokines in the udder and rapidly lose their functional features. The immune activity of MEC is regulated and balanced within the udder by factors locally delivered from other cell types [14]. These observations reinforce the interest of studying MEC biology in its physiological context, which can be done using RNA extracted from MFG. This non-invasive technique allowed analyzing the in vivo contribution of MEC during the very first steps of infection on the same individual, thus allowing a study of time-course variations while accounting for individual variability.

On the contrary, increased expression of genes encoding molecules with bactericidal functions, such as S100A12 and PTX3, is the second very clearly identified immune function of MEC. *PTX3* together with *S100A12* that could assist defense of the mammary gland against chronic and subclinical infections, have also been reported to be up-regulated in milk somatic cells in response to *S. aureus* infection in goats [54] and the resulting proteins were shown to be present in bovine milk, helping to resolve the mammary tissue infection as well as potentially contributing to the maturation of the newborn calf epithelium and establishment of the newborn gut microbial population [42]. Concomitantly with the early induction of cytokine and chemokine-encoding genes, we identified an up-regulation of two genes coding for acute phase proteins (APP): pentraxin 3 (PTX3) and α-1 anti-proteinase (SERPINA1) which were also found in bovine milk at the beginning of mastitis [39,42]. We demonstrate here from LCM experiments that MEC contribute significantly to the increases of PTX3 and SERPINA1 transcripts, even after the burst of immune cells in milk. Therefore, as previously proposed for SERPINA1 [55,56] which is a potent inhibitor of IL-8-induced hematopoietic stem cell mobilization [57], these two acute phase proteins could be useful markers in the early diagnosis of inflammation. We also observed a slight up-regulation of the gene encoding antimicrobial peptide S100A12 which is known to be induced by inflammatory cytokines such as IL-1β [58] and which demonstrates chemotactic activity, attracting circulating leucocytes in inflammation conditions [59]. This protein, which probably amplifies the inflammatory response via a recruitment of neutrophils [42], was reported to occur during all but the early stage response [60]. This is in agreement with our results. Indeed, it appears that MEC do not greatly contribute to its expression in the early stages of infection. However, the time-course of its response showed a clear tendency towards up-regulation from 24 hpi. Conversely, when the infection is well established, MEC are able to specifically express the acute phase protein SAA3 which can act directly on pathogens [61]. The gene encoding SAA3 is mainly induced by IL1-β and TNFα [62]. Studies have shown a growing interest for this protein and propose SAA3 as a good marker for mastitis. Interestingly, a high level of SAA has been observed in milk, in clinical [63] as well as in sub-clinical [64] bovine mastitis. Furthermore, whatever the dose and the invading pathogen, in vitro [65] as well as in vivo [17] studies reported high levels of mRNA molecules encoding SAA3 in response to infection or experimental challenge with *E. coli* or *S. aureus* cell wall components. However, 30 hpi we observed a very weakly (2.7 to 3-fold) enhanced expression of *SAA3*. This discrepancy could be explained, either by the fact that MEC in culture do not properly reflect MEC in their physiological context and/or that the udder is a complex tissue in which the dynamics of inflammation are different from that observed with a cellular model or even because the reading windows are not comparable. In previous studies (unpublished results), in agreement with the results reported by Eckersall et al. [64] in cattle, we observed that increases in acute phase SAA in milk of experimentally infected ewes occurred within 12 h after *S. aureus* infusion reaching a peak concentration at 72 h. Thirty hpi can still be considered as the onset of the IMI challenge with *S. aureus*, and consistent with our results, Eckersall et al. [64] who recorded at the early stage of infection (48 h) the expression of *SAA3* (mRNA), reported a slight increase (3.6-fold) in abundance of mRNA molecules. To substantiate our results, it is worth noting that most of these proteins were found in proteomic studies performed from *E. coli*, *S. aureus*[66] and *S. uberis*[67] mastitis milk whey. In addition, it should be underscored that the pattern of the response to IMI is basically similar and the qualitative differences observed between species most likely reflect different basal conditions and the type of invading pathogen [68].

Finally, as previously demonstrated in an in vitro study [12], we confirmed in vivo that the expression of *TLR2*, which is a key component in immune recognition of gram-positive bacteria by host cells, recognizing a wide spectrum of microbial components [69], was slightly affected by the infection (2.9-fold up-regulated 30 hpi) and essentially expressed by MEC (80% contribution). Thus, it is likely that MEC contain a fully functional and constitutively active Toll-Like Receptor signalling pathway that is slightly induced by the bacterial challenge but immediately responsive. Therefore, MEC have an intrinsic role in innate immune surveillance of the mammary tissue. The primary function is recruiting immune cells and the recognition likely occurs via the MyD88-dependent TLR signaling pathway. Stimulation of this pathway is considered the main mechanism enhancing expression of pro-inflammatory cytokines and activation of the innate immune response [70]. *TLR2* stimulation triggers intracellular signalling cascades leading to the activation of *NFκB* which, in turn, leads to the activation of several genes encoding pro-inflammatory mediators such as TNFα, IL-1β and IL-6. In addition, *TLR2* stimulation engages the production and release by MEC of chemokines such as IL-8, a potent chemo-attractant and activating factor of neutrophils, and, to a less extent, of cytokines CCL2 and CCL4 which display chemotaxis activity for monocytes and macrophages. Interestingly, due to a considerable among-goat variation, a number of genes possibly involved in MEC response to IMI challenge with *S. aureus*, has not emerged from the microarray analysis. Therefore, we analyzed some relevant genes, known to play crucial roles in the immune function, by comparing their gene expression profiles between goats, during the course of the infection. Thus, MyD88, an adapter protein involved in the Toll-Like Receptor and IL-1R signaling pathway, with a sharp increase at 24 h seemed to follow the same dynamic trends (Figure 9) as S100A12, whereas NFκB and IRAK4 (essential in the activation of NFκB), showed a strong increase at 18 hpi, before rapidly declining at 24 hpi, at least for 3 of the 5 goats analyzed in this study.

These data collectively demonstrate that we can easily access MEC biology in its physiological context via MFG. This non-invasive technique has allowed assessing the contribution of MEC during the first steps of infection, in vivo. Our results suggest that the MEC response to IMI challenge involves both genes likely to affect pathogen function as well as genes (e.g. cytokines) that alter the behaviour of other cell types. Consistent with others, the picture emerging from this study is that the mammary epithelium is not simply a mechanical barrier but rather a functionally complex tissue capable of responding effectively to the intrusion of pathogens by altering its own gene expression profile and possibly that of other cell types to favour recruitment of immune cells and to synthesize bactericidal molecules. We report here a novel strategy of sampling to monitor the dynamics of gene expression in MEC which allows going further into the understanding of MEC immune capacity. Furthermore, modulation of these roles could be of importance in determining the outcome of an infection. The chronologically induced synthesis of cytokines at the inflammation site is important for pathogen clearance, wound healing and return to normal conditions. This approach could allow a better understanding of MEC functions in animals showing different levels of genetic predisposition to mastitis, as tested with an in vitro study on sheep [71]. From a diagnosis point of view, several candidates for an early detection of mastitis were found.

Despite incomplete and imperfect annotation (the probe with the highest differential expression unfortunately remains unknown), which may explain why some effectors known to be induced in response to infection were not identified in this study (e.g. epithelial β-defensins such as LAP of which the expression is delayed [72] as well as other antibacterial molecules), the repertoire of gene probes used (Sheep Gene Expression Microarray, 8 × 15 K) has nevertheless confirmed the involvement of a number of master cytokines and chemokines (IL-8, IL-1β). Regarding IL-1β, which is first synthesized as biologically inactive pro-IL-1β, its processing into mature, biologically active pro-inflammatory cytokine supposes activation by caspase-1 which remains to be reported for MEC, before being released in the extracellular milieu following a non-classical secretory pathway [73]. One cannot take for granted that mRNA detection guaranties protein expression. The application of an original algorithm to construct gene networks of temporal regulation revealed the involvement of several factors known to play an important role in the inflammatory response, but for which we did not suspect that the MEC could be providers. In a recent review [74] and two proteomic studies [75,76], increasing of low abundant proteins such as IL-8, CD14, SAA, S100A12 and PTX3, were reported in mastitis milk from ewes and cows experimentally infected by *E. coli*, *S. aureus* and *S. uberis* or challenged with LPS, thus corroborating our transcriptional findings. Finally, the non-invasive sampling method (RNA extraction from MFG) provided an opportunity to perform a dynamic study of IMI, and this in spite of the significant individual variability observed which can in such a manner be partly bypassed. It also gives the opportunity to achieve a large-scale validation of the results in a significant number of individuals, which we plan to do in the near future.

## Competing interests

The authors declare that they have no competing interests.

## Authors’ contributions

PB was involved in design of study, carried out RNA extractions, performed microarray analyses, participated in statistical and bioinformatic analyses and drafted the manuscript. LL contributed to design and performed qPCR experiments. AR contributed to the statistical analyses of microarray data, constructed gene networks of temporal regulation and revised the manuscript critically. DL performed the statistical analyses of the microarray data. PMo and GP were responsible for the experimental *S. aureus* challenge of goats and sample collection and contributed to critical reading of the manuscript. CB participated in study design, performed Laser Capture Microdissection experiments, RNA extraction and drafted the manuscript. PMa conceived the study and its design, ensured coordination, participated in interpretation of data and contributed in writing the manuscript. All authors read and approved the final manuscript.

## References

[B1] Sordillo LM Streicher KL Mammary gland immunity and mastitis susceptibility J Mammary Gland Biol Neoplasia 2002 7 135 146 10.1023/A:1020347818725 12463736

[B2] Bannerman DD Pathogen-dependent induction of cytokines and other soluble inflammatory mediators during intramammary infection of dairy cows J Anim Sci 2009 87 Suppl13 10 25 18708595 10.2527/jas.2008-1187

[B3] Sordillo LM Shafer-Weaver K DeRosa D Immunobiology of the mammary gland J Dairy Sci 1997 80 1851 1865 10.3168/jds.S0022-0302(97)76121-6 9276826

[B4] Burton JL Erskine RJ Immunity and mastitis. Some new ideas for an old disease Vet Clin North Am Food Anim Pract 2003 19 1 45 12682934 10.1016/s0749-0720(02)00073-7

[B5] Rainard P Riollet C Innate immunity of the bovine mammary gland Vet Res 2006 37 369 400 10.1051/vetres:2006007 16611554

[B6] Bannerman DD Paape MJ Lee JW Zhao X Hope JC Rainard P *Escherichia coli* and *Staphylococcus aureus* elicit differential innate immune responses following intramammary infection Clin Diagn Lab Immunol 2004 11 463 472 15138171 10.1128/CDLI.11.3.463-472.2004 PMC404560

[B7] Paape MJ Shafer-Weaver K Capuco AV Oostveldt K Burvenich C **Immune surveillance of mammary tissue by phagocytic cell**s Adv Exp Med Biol 2002 480 259 277 10.1007/0-306-46832-8_31 10959434

[B8] Leitner G Eligulashvily R Krifucks O Perl S Saran A Immune cell differentiation in mammary gland tissues and milk of cows chronically infected with *Staphylococcus aureus* J Vet Med B Infect Dis Vet Public Health 2003 50 45 52 10.1046/j.1439-0450.2003.00602.x 12710501

[B9] Gray C Strandberg Y Donaldson L Tellam RL Bovine mammary epithelial cells, initiators of innate immune responses to mastitis Aust J Exp Agric 2005 45 757 762 10.1071/EA05046

[B10] Lahouassa H Moussay E Rainard P Riollet C Differential cytokine and chemokine responses of bovine mammary epithelial cells to *Staphylococcus aureus* and *Escherichia coli* Cytokine 2007 38 12 21 10.1016/j.cyto.2007.04.006 17532224

[B11] Swanson KM Stelwagen K Dobson J Henderson HV Davis SR Farr VC Singh K Transcriptome profiling of *Streptococcus uberis*-induced mastitis reveals fundamental differences between immune gene expression in the mammary gland and in a primary cell culture model J Dairy Sci 2009 92 117 129 10.3168/jds.2008-1382 19109270

[B12] Strandberg Y Gray C Vuocolo T Donaldson L Broadway M Tellam R Lipopolysaccharide and lipoteichoic acid induce different innate immune responses in bovine mammary epithelial cells Cytokine 2005 31 72 86 10.1016/j.cyto.2005.02.010 15882946

[B13] Pareek R Wellnitz O Van Dorp R Burton J Kerr D Immunorelevant gene expression in LPS-challenged bovine mammary epithelial cells J Appl Genet 2005 46 171 177 15876684

[B14] Gunther J Koczan D Yang W Nurnberg G Repsilber D Schuberth HJ Park Z Maqbool N Molenaar A Seyfert HM Assessment of the immune capacity of mammary epithelial cells: comparison with mammary tissue after challenge with *Escherichia coli* Vet Res 2009 40 31 10.1051/vetres/2009014 19321125 PMC2695127

[B15] Gunther J Esch K Poschadel N Petzl W Zerbe H Mitterhuemer S Blum H Seyfert HM Comparative kinetics of *Escherichia coli*- and *Staphylococcus aureus*-specific activation of key immune pathways in mammary epithelial cells demonstrates that *S. aureus* elicits a delayed response dominated by interleukin-6 (IL-6) but not by IL-1A or tumor necrosis factor alpha Infect Immun 2011 79 695 707 10.1128/IAI.01071-10 21115717 PMC3028868

[B16] Brenaut P Bangera R Bevilacqua C Rebours E Cebo C Martin P Validation of RNA isolated from milk fat globules to profile mammary epithelial cell expression during lactation and transcriptional response to a bacterial infection J Dairy Sci 2012 95 6130 6144 10.3168/jds.2012-5604 22921620

[B17] Zheng J Watson AD Kerr DE Genome-wide expression analysis of lipopolysaccharide-induced mastitis in a mouse model Infect Immun 2006 74 1907 1915 10.1128/IAI.74.3.1907-1915.2006 16495566 PMC1418644

[B18] Moroni P Pisoni G Vimercati C Rinaldi M Castiglioni B Cremonesi P Boettcher P Characterization of *Staphylococcus aureus* isolated from chronically infected dairy goats J Dairy Sci 2005 88 3500 3509 10.3168/jds.S0022-0302(05)73035-6 16162524

[B19] Wenz JR Barrington GM Garry FB Dinsmore RP Callan RJ Use of systemic disease signs to assess disease severity in dairy cows with acute coliform mastitis J Am Vet Med Assoc 2001 218 567 572 10.2460/javma.2001.218.567 11229511

[B20] Bevilacqua C Makhzami S Helbling JC Defrenaix P Martin P Maintaining RNA integrity in a homogeneous population of mammary epithelial cells isolated by Laser Capture Microdissection BMC Cell Biol 2010 11 95 10.1186/1471-2121-11-95 21134253 PMC3019183

[B21] Schroeder A Mueller O Stocker S Salowsky R Leiber M Gassmann M Lightfoot S Menzel W Granzow M Ragg T The RIN: an RNA integrity number for assigning integrity values to RNA measurements BMC Mol Biol 2006 7 3 10.1186/1471-2199-7-3 16448564 PMC1413964

[B22] SIGENAE [http://www.sigenae.org/], sheep oligo annotation, version 6 (2010/06/14)

[B23] Rau A Jaffrézic F Foulley JL Doerge RW An empirical Bayesian method for estimating biological networks from temporal microarray data Stat Appl Genet Mol Biol 2010 9 Article 9 10.2202/1544-6115.1513 20196759

[B24] Bevilacqua C Helbling JC Miranda G Martin P Translational efficiency of casein transcripts in the mammary tissue of lactating ruminants Reprod Nutr Dev 2006 46 567 578 10.1051/rnd:2006028 17107646

[B25] Hellemans J Mortier G De Paepe A Speleman F Vandesompele J qBase relative quantification framework and software for management and automated analysis of real-time quantitative PCR data Genome Biol 2007 8 R19 10.1186/gb-2007-8-2-r19 17291332 PMC1852402

[B26] Bustin SA Benes V Garson JA Hellemans J Huggett J Kubista M Mueller R Nolan T Pfaffl MW Shipley GL Vandesompele J Wittwer CT The MIQE guidelines: minimum information for publication of quantitative real-time PCR experiments Clin Chem 2009 55 611 622 10.1373/clinchem.2008.112797 19246619

[B27] Vandesompele J De Preter K Pattyn F Poppe B Van Roy N De Paepe A Speleman F Accurate normalization of real-time quantitative RT-PCR data by geometric averaging of multiple internal control genes Genome Biol 2002 3 RESEARCH0034 10.1186/gb-2002-3-7-research0034 PMC126239 12184808

[B28] Benjamini Y Hochberg Y Controlling the false discovery rate: a practical and powerful approach to multiple testing J R Statist Soc 1995 57 289 300

[B29] Petzl W Zerbe H Gunther J Yang W Seyfert HM Nurnberg G Schuberth HJ *Escherichia coli*, but not *Staphylococcus aureus* triggers an early increased expression of factors contributing to the innate immune defense in the udder of the cow Vet Res 2008 39 18 10.1051/vetres:2007057 18258172

[B30] Riollet C Rainard P Poutrel B Differential induction of complement fragment C5a and inflammatory cytokines during intramammary infections with *Escherichia coli* and *Staphylococcus aureus* Clin Diagn Lab Immunol 2000 7 161 167 10702487 10.1128/cdli.7.2.161-167.2000 PMC95843

[B31] Vangroenweghe F Duchateau L Burvenich C Moderate inflammatory reaction during experimental *Escherichia coli* mastitis in primiparous cows J Dairy Sci 2004 87 886 895 10.3168/jds.S0022-0302(04)73233-6 15259223

[B32] Bonnefont CM Toufeer M Caubet C Foulon E Tasca C Aurel MR Bergonier D Boullier S Robert-Granié C Foucras G Rupp R Transcriptomic analysis of milk somatic cells in mastitis resistant and susceptible sheep upon challenge with *Staphylococcus epidermidis* and *Staphylococcus aureus* BMC Genomics 2011 12 208 10.1186/1471-2164-12-208 21527017 PMC3096985

[B33] Riollet C Rainard P Poutrel B Cells and cytokines in inflammatory secretions of bovine mammary gland Adv Exp Med Biol 2002 480 247 258 10.1007/0-306-46832-8_30 10959433

[B34] Yang W Zerbe H Petzl W Brunner RM Günther J Draing C von Aulock S Schuberth HJ Seyfert HM Bovine TLR2 and TLR4 properly transduce signals from *Staphylococcus aureus* and *E. coli*, but *S. aureus* fails to both activate NF-kappaB in mammary epithelial cells and to quickly induce TNFalpha and interleukin-8 (CXCL8) expression in the udder Mol Immunol 2008 45 1385 1397 10.1016/j.molimm.2007.09.004 17936907

[B35] Gunther J Liu S Esch K Schuberth HS Seyfert HM Stimulated expression of TNF-alpha and IL-8, but not of lingual antimicrobial peptide reflects the concentration of pathogens contacting bovine mammary epithelial cells Vet Immunol Immunopathol 2010 135 152 157 10.1016/j.vetimm.2009.11.004 19963279

[B36] Maningat PD Sen P Rijnkels M Sunehag AL Hadsell DL Bray M Haymond MW Gene expression in the human mammary epithelium during lactation: the milk fat globule transcriptome Physiol Genomics 2009 37 12 22 10.1152/physiolgenomics.90341.2008 19018045 PMC2661101

[B37] Moyes KM Drackley JK Morin DE Bionaz M Rodriguez-Zas SL Everts RE Lewin HA Loor JJ Gene network and pathway analysis of bovine mammary tissue challenged with *Streptococcus uberis* reveals induction of cell proliferation and inhibition of PPARgamma signaling as potential mechanism for the negative relationships between immune response and lipid metabolism BMC Genomics 2009 10 542 10.1186/1471-2164-10-542 19925655 PMC2784807

[B38] Barber MR Pantschenko AG Hinckley LS Yang TJ Inducible and constitutive *in vitro* neutrophil chemokine expression by mammary epithelial and myoepithelial cells Clin Diagn Lab Immunol 1999 6 791 798 10548565 10.1128/cdli.6.6.791-798.1999 PMC95777

[B39] Mitterhuemer S Petzl W Krebs S Mehne D Klanner A Wolf E Zerbe H Blum H *Escherichia coli* infection induces distinct local and systemic transcriptome responses in the mammary gland BMC Genomics 2010 11 138 10.1186/1471-2164-11-138 20184744 PMC2846913

[B40] Jensen K Günther J Talbot R Petzl W Zerbe H Schuberth HJ Seyfert HM Glass EJ Escherichia coli- and Staphylococcus aureus-induced mastitis differentially modulate transcriptional responses in neighbouring uninfected bovine mammary gland quarters BMC Genomics 2013 14 36 10.1186/1471-2164-14-36 23324411 PMC3598231

[B41] Le Gall A Plommet M Observations sur la croissance des staphylocoques et la réaction leucocytaire au cours des premières heures de la mammite expérimentale de la brebis Ann Biol Anim Bioch Biophys 1965 5 113 130 (in French) 10.1051/rnd:19650108

[B42] Lutzow YC Donaldson L Gray CP Vuocolo T Pearson RD Reverter A Byrne KA Sheehy PA Windon R Tellam RL Identification of immune genes and proteins involved in the response of bovine mammary tissue to *Staphylococcus aureus* infection BMC Vet Res 2008 4 18 10.1186/1746-6148-4-18 18513449 PMC2430192

[B43] Salvador JM Mittelstadt PR Belova GI Fornace AJ Ashwell JD The autoimmune suppressor Gadd45alpha inhibits the T cell alternative p38 activation pathway Nat Immunol 2005 6 396 402 10.1038/ni1176 15735649

[B44] Dostaler-Touchette V Bédard F Guillemette C Pothier F Chouinard PY Richard FJ Cyclic adenosine monophosphate (cAMP)-specific phosphodiesterase is functional in bovine mammary gland J Dairy Sci 2009 92 3757 3765 10.3168/jds.2009-2065 19620657

[B45] Ariga M Neitzert B Nakae S Mottin G Bertrand C Pruniaux MP Jin SL Conti M Nonredundant function of phosphodiesterases 4D and 4B in neutrophil recruitment to the site of inflammation J Immunol 2004 173 7531 7538 15585880 10.4049/jimmunol.173.12.7531

[B46] Jin SL Lan L Zoudilova M Conti M Specific role of phosphodiesterase 4B in lipopolysaccharide-induced signaling in mouse macrophages J Immunol 2005 175 1523 1531 16034090 10.4049/jimmunol.175.3.1523

[B47] Fitzgerald DC Meade KG McEvoy AN Lillis L Murphy EP MacHugh DE Baird AW Tumour necrosis factor-alpha (TNF-alpha) increases nuclear factor kappa B (NF kappa B) activity in and interleukin-8 (IL-8) release from bovine mammary epithelial cells Vet Immunol Immunopathol 2007 116 59 68 10.1016/j.vetimm.2006.12.008 17276517

[B48] Persson Waller K Colditz IG Lun S Ostensson K Cytokines in mammary lymph and milk during endotoxin-induced bovine mastitis Res Vet Sci 2003 74 31 36 12507564 10.1016/s0034-5288(02)00147-9

[B49] Pisoni G Moroni P Genini S Stella A Boettcher PJ Cremonesi P Scaccabarozzi L Giuffra E Castiglioni B Differentially expressed genes associated with *Staphylococcus aureus* mastitis in dairy goats Vet Immunol Immunopathol 2010 135 208 217 10.1016/j.vetimm.2009.11.016 20060596

[B50] Le Provost F MiYoshi K Vilotte JL Briere B Robinson GW Hennighausen L SOCS3 promotes apoptosis of mammary differentiated cells Biochem Biophys Res Com 2005 338 1696 1701 10.1016/j.bbrc.2005.10.138 16289036

[B51] Robinson GW Pacher-Zavisin M Zhu BM Yoshimura A Hennighausen L Socs3 modulates the activity of the transcription factor Stat3 in mammary tissue and controls alveolar homeostasis Dev Dyn 2007 236 654 661 10.1002/dvdy.21058 17205581

[B52] Heeg K Dalpke A TLR-induced negative regulatory circuits: role of suppressorof cytokine signaling (SOCS) proteins in innate immunity Vaccine 2003 21 Suppl 2 S61 67 12763685 10.1016/s0264-410x(03)00202-0

[B53] Croker BA Kiu H Nicholson SE SOCS regulation of the JAK/STAT signalling pathway Semin Cell Dev Biol 2008 19 414 422 10.1016/j.semcdb.2008.07.010 18708154 PMC2597703

[B54] Cremonesi P Capoferri R Pisoni G Del Corvo M Strozzi F Rupp R Caillat H Modesto P Moroni P Williams JL Castiglioni B Stella A Response of the goat mammary gland to infection with *Staphylococcus aureus* revealed by gene expression profiling in milk somatic and white blood cells BMC Genomics 2012 13 540 10.1186/1471-2164-13-540 23046560 PMC3532242

[B55] Huszenicza G Kégl T Kulcsar M Olah B Gacs M Oppel K Stollar Z Jonsson P Janosi S Diagnostic value of certain mastitis markers in following up the clinical and bacteriological changes in pharmacotherapeutic studies Acta Vet Hung 1997 45 409 416 9557318

[B56] Boehmer JL Bannerman DD Shefcheck K Ward JL Proteomic analysis of differentially expressed proteins in bovine milk during experimentally induced *Escherichia coli* mastitis J Dairy Sci 2008 91 4206 4218 10.3168/jds.2008-1297 18946125

[B57] van Pel M van Os R Velders GA Hagoort H Heegaard PM Lindley IJ Willemze R Fibbe WE Serpina1 is a potent inhibitor of IL-8-induced hematopoietic stem cell mobilization Proc Natl Acad Sci USA 2006 103 1469 1474 10.1073/pnas.0510192103 16432201 PMC1360568

[B58] Kolls JK McCray PB Jr Chan YR Cytokine-mediated regulation of antimicrobial proteins Nat Rev Immunol 2008 8 829 835 10.1038/nri2433 18949018 PMC2901862

[B59] Pietzsch J Hoppmann S Human S100A12: a novel key player in inflammation? Amino Acids 2009 36 381 389 10.1007/s00726-008-0097-7 18443896

[B60] Genini S Badaoui B Sclep G Bishop SC Waddington D van der Laan M-H P Klopp C Cabau C Seyfert HM Petzl W Jensen K Glass EJ de Greeff A Smith HE Smits MA Olsaker I Boman GM Pisoni G Moroni P Castiglioni B Cremonesi P Del Corvo M Foulon E Foucras G Rupp R Giuffra E Strengthening insights into host responses to mastitis infection in ruminants by combining heterogeneous microarray data sources BMC Genomics 2011 12 225 10.1186/1471-2164-12-225 21569310 PMC3118214

[B61] Molenaar AJ Harris DP Rajan GH Pearson ML Callaghan MR Sommer L Farr VC Oden KE Miles MC Petrova RS Good LL Singh K McLaren RD Prosser CG Kim KS Wieliczko RJ Dines MH Johannessen KM Grigor MR Davis SR Stelwagen K The acute-phase protein serum amyloid A3 is expressed in the bovine mammary gland and plays a role in host defence Biomarkers 2009 14 26 37 10.1080/13547500902730714 19283521

[B62] Jensen LE Whitehead AS Regulation of serum amyloid A protein expression during the acute-phase response Biochem J 1998 334 489 503 9729453 10.1042/bj3340489 PMC1219714

[B63] Jacobsen S Niewold TA Kornalijnslijper E Toussaint MJ Gruys E Kinetics of local and systemic isoforms of serum amyloid A in bovine mastitic milk Vet Immunol Immunopathol 2005 104 21 31 10.1016/j.vetimm.2004.09.031 15661328

[B64] Eckersall PD Young FJ Nolan AM Knight CH McComb C Waterston MM Hogarth CJ Scott EM Fitzpatrick JL Acute phase proteins in bovine milk in an experimental model of *Staphylococcus aureus* subclinical mastitis J Dairy Sci 2006 89 1488 1501 10.3168/jds.S0022-0302(06)72216-0 16606719

[B65] Weber A Weber AT McDonald TL Larson MA *Staphylococcus aureus* lipotechoic acid induces differential expression of bovine serum amyloid A3 (SAA3) by mammary epithelial cells: implications for early diagnosis of mastitis Vet Immunol Immunopathol 2006 109 79 83 10.1016/j.vetimm.2005.07.023 16139367

[B66] Ibeagha-Awemu EM Ibeagha AE Messier S Zhao X Proteomics, genomics and pathway analyses of *Escherichia coli* and *Staphylococcus aureus* infected milk whey reveal molecular pathways and networks involved in mastitis J Proteome Res 2010 9 4604 4619 10.1021/pr100336e 20704270

[B67] Smolenski G Haines S Kwan FY Bond J Farr V Davis SR Stelwagen K Wheeler TT Characterisation of host defence proteins in milk using a proteomic approach J Proteome Res 2007 6 207 215 10.1021/pr0603405 17203965

[B68] Leitner G Merin U Krifucks O Blum S Rivas AL Silanikove N Effects of intra-mammary bacterial infection with coagulase negative staphylococci and stage of lactation on shedding of epithelial cells and infiltration of leukocytes into milk: comparison among cows, goats and sheep Vet Immunol Immunopathol 2012 147 202 210 10.1016/j.vetimm.2012.04.019 22584045

[B69] Kumar H Kawai T Akira S Pathogen recognition by the innate immune system Int Rev Immunol 2011 30 16 34 10.3109/08830185.2010.529976 21235323

[B70] Takeda K Akira S Toll-like receptors in innate immunity Int Immunol 2005 17 1 14 15585605 10.1093/intimm/dxh186

[B71] Bonnefont CM Rainard P Cunha P Gilbert FB Toufeer M Aurel MR Rupp R Foucras G Genetic susceptibility to *S. aureus* mastitis in sheep: differential expression of mammary epithelial cells in response to live bacteria or supernatant Physiol Genomics 2012 44 403 416 10.1152/physiolgenomics.00155.2011 22337903

[B72] Liu S Shi X Bauer I Günther J Seyfert HM Lingual antimicrobial peptide and IL-8 expression are oppositely regulated by the antagonistic effects of NF-κB p65 and C/EBPβ in mammary epithelial cells Mol Immunol 2011 48 895 908 10.1016/j.molimm.2010.12.018 21255844

[B73] Eder C Mechanisms of interleukin-1β release Immunobiology 2009 214 543 553 10.1016/j.imbio.2008.11.007 19250700

[B74] Boehmer JL Proteomic analyses of host and pathogen responses during bovine mastitis J Mammary Gland Biol Neoplasia 2011 16 323 338 10.1007/s10911-011-9229-x 21892748 PMC3208817

[B75] Kim Y Atalla H Mallard B Robert C Karrow N Changes in Holstein cow milk and serum proteins during intramammary infection with three different strains of *Staphylococcus aureus* BMC Vet Res 2011 7 51 10.1186/1746-6148-7-51 21884610 PMC3179444

[B76] Addis MF Pisanu S Marogna G Cubeddu T Pagnozzi D Cacciotto C Campesi F Schianchi G Rocca S Uzzau S Production and release of antimicrobial and immune defense proteins by mammary epithelial cells following *Streptococcus uberis* infection of sheep Infect Immun 2013 81 3182 3197 10.1128/IAI.00291-13 23774600 PMC3754230

